# Cation‐Site Disordered Cu_3_PdN Nanoparticles for Hydrogen Evolution Electrocatalysis

**DOI:** 10.1002/smll.202506838

**Published:** 2025-06-20

**Authors:** Sani Y. Harouna‐Mayer, Jagadesh Kopula Kesavan, Francesco Caddeo, Lian Belgardt, Chia‐Shuo Hsu, Lars Klemeyer, Lizzi Kipping, Melike Gumus Akcaalan, Tjark R.L. Groene, Andrea Köppen, Heshmat Noei, Olivier Mathon, Ann‐Christin Dippel, Dorota Koziej

**Affiliations:** ^1^ Institute for Nanostructure and Solid‐State Physics Center for Hybrid Nanostructures (CHyN) University of Hamburg 22761 Hamburg Germany; ^2^ The Hamburg Center for Ultrafast Imaging 22761 Hamburg Germany; ^3^ Center for X‐ray and Nano Science CXNS Deutsches Elektronen‐Synchrotron DESY 22607 Hamburg Germany; ^4^ Department of Chemistry University of Hamburg 20146 Hamburg Germany; ^5^ European Synchrotron Radiation Facility (ESRF) Grenoble 38043 France; ^6^ Deutsches Elektronen‐Synchrotron DESY 22607 Hamburg Germany

**Keywords:** anti‐perovskite structure, cation disorder, double‐edge EXAFS refinement, hydrogen evolution reaction, in situ X‐ray absorption and scattering studies, ternary metal nitrides

## Abstract

Transition metal nitrides (TMNs) are emerging as a promising class of materials for application in optoelectronics as well as energy conversion and storage, but they remain rather unexplored, mainly due to a lack of mechanistic understanding of their synthetic pathways. Here, a one‐pot synthesis is demonstrated, which yields 3 nm phase‐pure Cu_3_PdN nanoparticles after the reaction of Cu methoxide and Pd acetylacetonate in benzylamine for 5 min at 140 °C. The structure of the initial complexes and their conversion to Cu_3_PdN are revealed by in situ X‐ray absorption spectroscopy measurements and elucidate nucleation and growth of the nitride nanocrystals by in situ total X‐ray scattering measurements. Interestingly, extended X‐ray absorption fine structure double‐edge refinement reveals the presence of short‐range cation‐site disorder in the anti‐perovskite structure of Cu_3_PdN, which has not been observed before in the Cu_3_PdN system. Additionally, the synthesized Cu_3_PdN nanoparticles are tested for the electrocatalytic hydrogen evolution reaction, revealing an overpotential as low as η_10_ = 212 ± 11 mV measured at 10 mA cm^−2^.

## Graphical abstract

The reaction of Cu methoxide with Pd acetylacetonate is reported, which yields 3 nm Cu_3_PdN nanoparticles after 5 min at 140 °C in benzylamine. The synthesized nanoparticles exhibit a short‐range cation‐site disorder and are further tested for the hydrogen evolution reaction. A comprehensive in situ X‐ray absorption and in situ total X‐ray scattering study reveal the reaction pathways of the reaction.

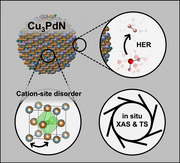

## 1 Introduction

Transition metal nitrides (TMNs) are a versatile class of materials that exhibit properties of covalent compounds, ionic crystals, and transition metals.^[^ ^1^, ^2^, ^3^ ^]^ The incorporation of the nitride (N^3−^) anion into the metal sublattice causes expansion of the parent metal lattice and metal d‐band broadening.^[^ ^4^, ^5^, ^6^ ^]^ These unique electronic and bonding characteristics give rise to numerous applications, particularly in energy conversion and storage.^[^ ^2^, ^3^, ^7^, ^8^, ^9^ ^]^ Recent studies explored several TMNs as materials of interest for electrochemical energy conversion, focusing on the influence of morphology, ^[^ ^10^, ^11^, ^12^ ^]^ defect engineering, ^[^ ^13^, ^14^, ^15^ ^]^ heteroatom doping, ^[^ ^16^, ^17^ ^]^ heterostructuring^[^ ^18^, ^19^ ^]^ or alloying^[^ ^20^, ^21^ ^]^ on their electrochemical performance. Despite the progress, challenges remain, such as achieving scalability, and stability in aqueous media, or cost‐effective and green synthetic methods, since TMN synthesis typically involves steps at high temperatures and/or pressure.^[^ ^8^, ^9^ ^]^ Further, the synthesis of TMNs involves reaction pathways that are still poorly understood. A deeper understanding of the synthesis and reaction mechanisms is crucial to fully explore the TMN space and develop TMN materials with desired chemical and physical properties with respect to their application.^[^ ^8^, ^22^, ^23^ ^]^

Among TMNs, Cu_3_N has emerged as a promising material since it is non‐toxic and earth‐abundant, showing potential for cost‐efficient solar energy conversion, catalysis, and optoelectronics.^[^ ^24^ ^]^ Cu_3_N exhibits the anti‐ReO_3_ structure with corner‐shared N‐centered Cu─N octahedra. The vacant cubic central position can be occupied by a dopant such as Pd, forming, e.g., Cu_3_PdN, which changes the electronic structure from semiconducting to semimetallic.^[^ ^25^, ^26^, ^27^, ^28^, ^29^ ^]^ Cu_3_PdN has been proposed to be a node‐line semimetal and topological superconductor.^[^ ^30^, ^31^, ^32^ ^]^ Further, Cu_3_PdN shows good electrochemical catalytic activity and stability for nitrate reduction, ^[^ ^33^ ^]^ CO_2_ reduction, ^[^ ^34^, ^35^ ^]^ O_2_ reduction^[^ ^36^ ^]^ and formic acid oxidation. ^[^ ^37^ ^]^ So far, there are no reports on its activity for the hydrogen evolution reaction (HER). Independent of the application, Cu_3_PdN exhibits superior catalytic activity and/or stability over the Pd‐deficient Cu_3_N^[^ ^33^, ^36^ ^]^ as well as N‐deficient Cu_3_Pd^[^ ^34^, ^37^ ^]^ or Pd^[^ ^33^, ^36^, ^37^ ^]^ counterparts.

All reported solvothermal synthetic approaches for Cu_3_PdN follow the same route: Cu(NO_3_)_2_·3H_2_O and Pd(acac)_2_ form 10 – 20 nm sized Cu_3_PdN nanoparticles (NPs) after 5–60 min at 230–250 °C in oleylamine (OAm) and 1‐octadecene or hexadecane (Table S1, Supporting Information).^[^ ^33^, ^34^, ^35^, ^36^, ^37^, ^38^ ^]^ The reaction temperature can be decreased to 190 °C if Au, Pt, and Pt‐Fe_3_O_4_ NP seeds are used.^[^ ^39^ ^]^ In the reported synthesis route, OAm is expected to be both a reductant and a nitrogen source. First OAm is oxidized by the nitrate and Cu(II) to a primary aldimine, which reacts with extant OAm to a secondary aldimine, releasing ammonia. Consequently, ammonia reacts with Cu(I), forming Cu_3_PdN.^[^ ^38^ ^]^ This mechanism has been proposed based on the analysis of the reaction byproducts using ^1^H nuclear magnetic resonance (NMR), which is sensitive to the organic species present at the end of the reaction but does not give exhaustive information on the nature of the metal complexes leading to the formation of the Cu_3_PdN NPs. Moreover, limited information is given on the phase purity of Cu_3_PdN NPs obtained with the OAm route, since the published powder X‐ray diffraction (PXRD) patterns display a peak shape anisotropy that likely indicates the presence of bimetallic Cu_3_Pd impurities.^[^ ^33^, ^34^, ^35^, ^36^, ^37^, ^38^, ^39^ ^]^

Here, we report a rapid, one‐pot synthesis route that yields phase‐pure, highly crystalline 3 nm Cu_3_PdN NPs. Unlike aforementioned reports on Cu_3_PdN synthesis,^[^ ^33^, ^34^, ^35^, ^36^, ^37^, ^38^, ^39^ ^]^ we can decrease the reaction temperature to 140 °C by replacing copper (II) nitrate trihydrate with copper (II) methoxide and dissolving it, jointly with palladium (II) acetylacetonate, in benzylamine instead of OAm. Similar to the Cu_3_N synthesis,^[^ ^40^ ^]^ benzylamine acts as a ligand, reductant, and nitrogen source.

First, we perform Rietveld refinement and extended X‐ray absorption fine structure (EXAFS) analysis of ex situ PXRD and X‐ray absorption spectroscopy (XAS) data, respectively, to demonstrate phase purity and to examine the local structure around Cu and Pd in the anti‐perovskite Cu_3_PdN crystal structure. We find that the as‐prepared 3 nm Cu_3_PdN NPs display cation‐site disorder, with the Cu and Pd atoms being distributed at the corners and at the face centers of the cation fcc sublattice, with partial occupancy. This is the first time that cation‐site disorder has been reported for the case of colloidal TMNs with the anti‐perovskite structure.^[^ ^41^, ^42^, ^43^ ^]^

Additionally, using in situ high‐energy resolution fluorescence detected X‐ray absorption near‐edge spectroscopy (HERFD‐XANES) and in situ pair distribution function (PDF) analysis of total scattering (TS), we investigate the reaction pathways involving the Cu and Pd starting complexes during the synthesis of Cu_3_PdN in benzylamine. HERFD‐XANES is a powerful, element‐specific technique that probes the emergence of the electronic structure of a material and monitors the coordination of metal centers during the synthesis, including the formation and transformation of metal‐organic complexes and intermediates into the desired crystalline nanomaterial, thereby shedding light on the reaction mechanism.^[^ ^44^, ^45^ ^]^ Meanwhile, PDF analysis investigates the atomic structure of a material and thus allows for monitoring of the nucleation and growth of Cu_3_PdN NPs.^[^ ^46^ ^]^ Hence, in situ HERFD‐XANES complemented by in situ PDF provides both electronic and structural insights, giving a comprehensive picture of the synthetic pathways together with the appearance of metallic Cu_3_Pd impurities for extended reaction times.

**Figure 1:**
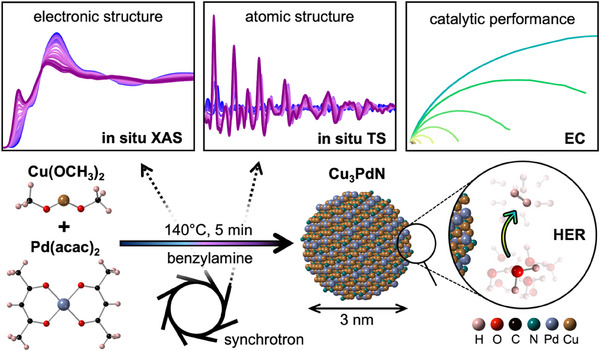
Schematic illustration of the present work. The reaction of Cu(II) methoxide and Pd(II) acetylacetonate, which yields Cu_3_PdN nanoparticles after 5 min at 140 °C in benzylamine, is studied using in situ X‐ray absorption spectroscopy (XAS) and in situ total scattering (TS). The synthesized Cu_3_PdN nanoparticles are tested for their electrochemical (EC) catalytic activity of the hydrogen evolution reaction (HER).

Finally, we test the electrocatalytic properties of the as‐synthesized Cu_3_PdN NPs for the HER, showing good catalytic activity with an overpotential of η_10_ = 212 ± 11 mV measured at 10 mA/cm^2^ and excellent stability with no change of the overpotential after 10 000 linear sweep voltammetry (LSV) cycles. **Figure** **1** shows a schematic representation of the work conducted in this report.

## 2 Results and Discussion

**Figure 2:**
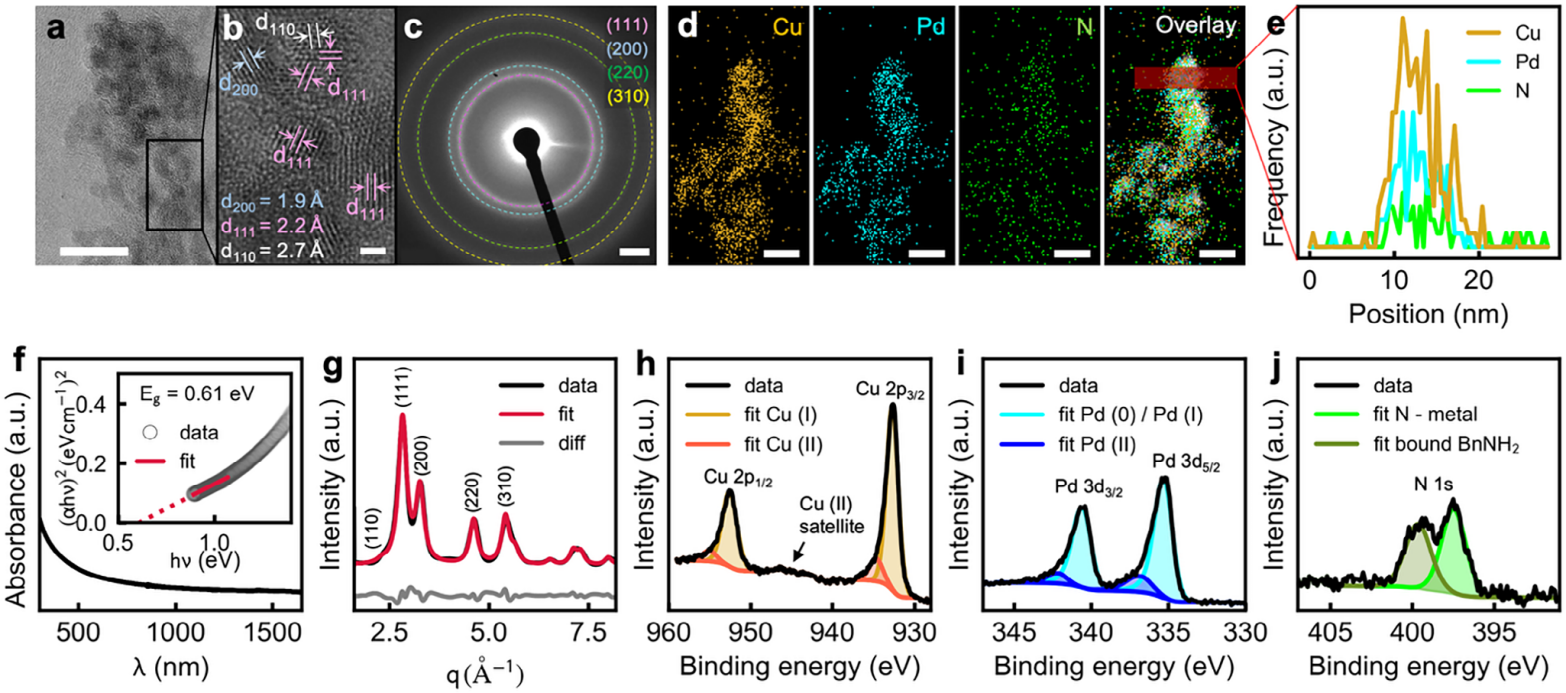
Ex situ characterization of Cu_3_PdN NPs grown after 5 min reaction time. a) HRTEM of Cu_3_PdN NP agglomerate. b) Zoom of a showing polydisperse spherical NPs. c) SAED of a Cu_3_PdN assembly. d) STEM:EDX elemental mapping of Cu, Pd, and N and an overlay of the mapped elements on the original STEM image. e) EDX elemental line profiles of the area highlighted in c showing evenly distributed Cu, Pd, and N. f) UV–vis absorption spectrum of Cu_3_PdN NPs redispersed in N‐methyl‐2‐pyrrolidone with its corresponding Tauc plot, assuming a direct allowed transition as an inset. The dotted red trace shows the extrapolation of the linear fit shown as a solid red trace. g) PXRD Rietveld refinement. XPS core‐level spectra of h Cu 2p, i Pd 3d, and j N 1s. Scales: a: 10 nm; b: 1 nm; c: 2 1/nm; d: 10 nm.

We perform the solvothermal synthesis of Cu_3_PdN nanoparticles by reacting Cu(OCH_3_)_2_ and Pd(acac)_2_ in benzylamine, which acts both as the solvent and nitrogen source. The reaction is carried out at 140 °C under an inert atmosphere in a custom‐made heating cell that enables precise control of reaction parameters such as the reaction temperature, reaction time, ramp rate, and stirring of the reaction solution.^[^ ^47^ ^]^ The reaction yields Cu_3_PdN nanoparticles with sizes of ∼3.5 nm for reaction times of 5 to 15 min, as shown in Figure S1 (Supporting Information). **Figure** **2**a shows a high‐resolution transmission electron microscopy (HRTEM) image of the Cu_3_PdN nanoparticles after 5 min of reaction time. The identified lattice spacing of 1.9, 2.2, and 2.7 Å corresponds to the (200), (111), and (110) planes, respectively, and are highlighted in Figure 2b. The corresponding selected area electron diffraction (SAED) pattern displays diffraction peaks with positions and intensities consistent with the anti‐perovskite crystal structure of Cu_3_PdN, as shown in Figure 2c. The elemental color mapping with overlay and line scan confirms that the nanoparticles are composed of Cu, Pd, and N atoms and their signals overlap uniformly as shown in Figure 2d,e. We determine the composition of the as‐synthesized nanoparticles by elemental analysis to be Cu_3.06_Pd_1.00_N_1.23_, consistent with the expected Cu_3_PdN stoichiometry, where the high nitrogen content is attributed to surface‐bound benzylamine. Figure 2f shows the UV–vis spectra of Cu_3_PdN nanoparticles. Unlike a previous study that reported an indirect bandgap of 0.2 eV, we observe a direct optical bandgap of 0.61 eV. The discrepancies may arise due to the particle size being four times smaller and, more importantly, the absence of impurities in our sample.^[^ ^38^ ^]^ Further studies of the optical properties are beyond the scope of this work. The thermogravimetric analysis (TGA) of the obtained Cu_3_PdN nanoparticles in nitrogen atmosphere (Figure S2, Supporting Information) depicts a weight loss at low temperature below 160 °C due to the removal of residual organics and a second one at temperatures above 250 °C likely due to the reduction of Cu_3_PdN to bimetallic/metallic.^[^ ^38^ ^]^

Cu_3_Pd might form as a secondary phase during the synthesis of Cu_3_PdN, thus we first check the phase purity of the obtained Cu_3_PdN nanoparticles via Rietveld refinement of the PXRD patterns, as shown in Figure 2g. The PXRD patterns of Cu_3_PdN and Cu_3_Pd are very similar, with subtle differences in peak intensity and position, since both structures share the same space group and exhibit a slightly different lattice parameter as illustrated in Figure S3 (Supporting Information). In Figure S4 (Supporting Information), we simulate two‐phase PXRD patterns with different phase ratios of Cu_3_PdN:Cu_3_Pd. The presence of a Cu_3_Pd impurity generates a peak‐shape anisotropy, which is most prominently visible at the most intense (111) reflection at ≈*2Θ* = 41° (assuming Cu K‐alpha radiation, *q* = 2.9 Å^−1^). This peak shape anisotropy is present in most of the PXRD patterns of previously reported Cu_3_PdN syntheses,^[^ ^33^, ^34^, ^35^, ^36^, ^37^, ^38^, ^39^ ^]^ confirming that the presence of Cu_3_Pd impurity is often overlooked. The PXRD patterns of our Cu_3_PdN nanoparticles prepared with a reaction time of 5, 10, and 15 min are reported in Figure S5 (Supporting Information)and confirm the absence of Cu_3_Pd impurities. The Cu_3_Pd phase appears only after reaction times longer than 15 min, as also confirmed by our in situ TS study discussed below.

The nitride phase purity of synthesized Cu_3_PdN NPs is further confirmed by the X‐ray photoelectron spectroscopy (XPS). The core level Cu 2p, Pd 3d, and N 1s spectra of synthesized Cu_3_PdN NPs are depicted in Figure 2h–j. The Cu binding energies (BE) of the main peaks at 932.69 and 952.54 eV are assigned to Cu 2p_3/2_ and Cu 2p_1/2,_ respectively, and these binding energies are related to the monovalent Cu (I) species.^[^ ^33^, ^34^, ^35^, ^36^, ^37^, ^38^ ^]^ In addition to the main Cu (I) peaks, a less intense peak at 934.66 and 955.03 eV and the presence of a very weak‐intense satellite peak ≈941–946 eV are related to Cu (II), which is found in all the other reports of Cu_3_PdN.^[^ ^33^, ^34^, ^35^, ^36^, ^37^, ^38^ ^]^ The Pd 3d_5/2_ and 3d_3/2_ core level components at 335.23 and 340.46 eV, respectively, are related to Pd (0)/Pd (I). The secondary weak intense peaks at 335.97 and 341.49 eV are related to the Pd (II).^[^ ^33^ ^]^ From the peak fitting analysis, the Cu (II) and Pd (II) contributions are ≈13.5% and 7.9%, respectively. The N 1s core level spectrum is deconvoluted into two peaks at 397.47 and 399.63 eV are related to the metal‐nitrogen bond and residual benzylamine bound to the surface of the Cu_3_PdN NPs, respectively.

To further confirm the phase purity, we perform EXAFS analysis, which allows to determine the local structure around the absorbing atom.^[^ ^48^ ^]^ In addition to being an element‐specific technique, EXAFS is well suited for multielement materials, allowing to obtain insights such as atomic rearrangements, defects, and disorders.

**Figure 3:**
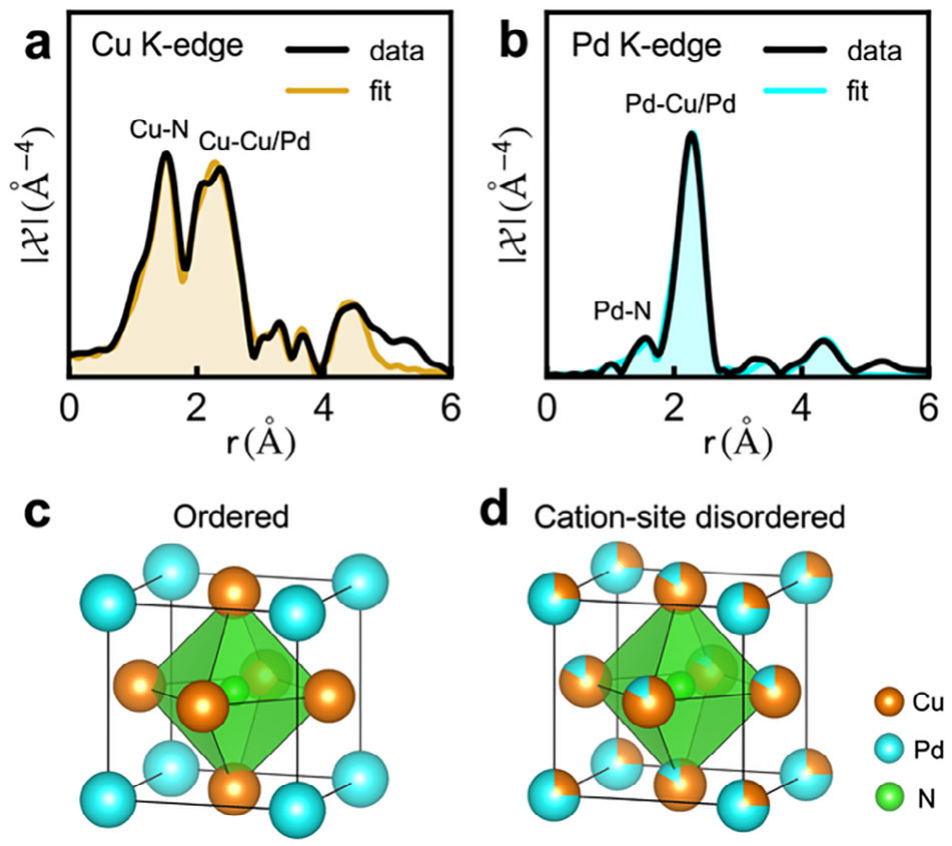
EXAFS fits showing the cation‐site disorder. a) Cu K‐edge EXAFS fit. b) Pd K‐edge EXAFS fit. c) Ordered and d) cation‐site disordered anti‐perovskite unit cell of Cu_3_PdN.

The attempt to refine the ex situ EXAFS data of both Cu and Pd K‐edges using the ordered anti‐perovskite crystal structure does not yield a satisfactory agreement, as shown in Figure S6 (Supporting Information). A careful look at the Fourier‐transformed Pd K‐edge data compared with the simulated spectrum (Figure S7, Supporting Information) reveals the presence of a peak ≈1.6 Å might be originating from the scattering contribution of Pd‐N distances in the first coordination shell of Pd atoms. This is a clear indication of the presence of a partial short‐range cation‐site disorder in the Cu_3_PdN NPs, where the Cu and Pd atoms are located at the corners and at the face centers of the cation fcc sublattice, with partial occupancy, as shown in Figure 2j. This result is further supported by the oxidation state of the Cu cation. In the ordered structure, the average oxidation state of Cu is +1^[^ ^38^ ^]^ and it is expected to be < +1 for the structure with cation‐site disorder. The maximum of the first derivative of Cu K‐edge XANES of Cu_3_PdN in Figure S8 (Supporting Information) is slightly higher (8979.7 eV) than the reference Cu foil (8979.0 eV) and lower than Cu_3_N (8980.28 eV). Furthermore, when compared to the literature value for Cu_3_N and Cu_2_O (8980.5 eV) in which the oxidation state of Cu is +1,^[^ ^49^, ^50^ ^]^ our sample's edge position is slightly at a lower energy (ΔE = 0.8 eV). This clearly emphasizes that the average oxidation state of Cu in Cu_3_PdN is < +1, which further hints at the presence of cation‐site disorder in our Cu_3_PdN NPs. We therefore perform a double‐edge EXAFS refinement, utilizing both Cu K‐edge and Pd K‐edge EXAFS data, which allows to resolve the local structure around the Cu and Pd atom and to quantitatively assess the cation‐site occupancy. The multi‐shell fitting method employed uses the physical constraints given by the crystallographic structure, which includes the lattice parameter and the Cu‐Pd and Pd‐Pd distances. The many‐body amplitude reduction factor ($S_{0}^{2}$) and the energy origin shift (ΔE_0_) were considered as fixed parameters.^[^ ^51^ ^]^ Further details of the EXAFS analysis are given in the supporting information. The Fourier‐transformed EXAFS spectra and their best fits are shown in **Figure** **3**a,b and in Figure S6 (Supporting Information). It is important to note and it is common that the cation‐site disorder induces a local distortion partially, leading to the short Cu─Pd bond length of 0.08–0.1 Å in the second shell without affecting the lattice symmetry.^[^ ^52^ ^]^ The EXFAS fit of our Cu_3_PdN NPs confirms the presence of partial short‐range cation‐site disorder, where ≈25% of the Pd atoms exchanged their lattice position. The interatomic distances, the disorder fraction (1‐x), and Debye‐Waller factors obtained from the fit can be found in Table S3 (Supporting Information). This short‐range cation‐site disorder occurs mainly due to low cation mobility, fast growth rate at low temperatures, and short reaction times employed during the synthesis, which favors the formation of the disordered over the ordered structures.^[^ ^53^ ^]^

The short‐ and long‐range disorders have already been experimentally observed and theoretically predicted in II‐IV‐N_2_ wurtzite‐derived structures^[^ ^43^, ^54^, ^55^, ^56^, ^57^ ^]^ (e.g., MgSnN_2_, ZnGeN_2_) and other TMNs.^[^ ^58^, ^59^, ^60^ ^]^ The cation‐site disorder in the nitride system is highly beneficial and allows to control and tune the properties such as band gap, ion, and thermal conductivity.^[^ ^61^, ^62^ ^]^ Nevertheless, it is important to highlight that this type of cation‐site disorder has not been reported before for the case of colloidal anti‐perovskite structured nitride systems, especially in Cu_3_PdN.

**Figure 4:**
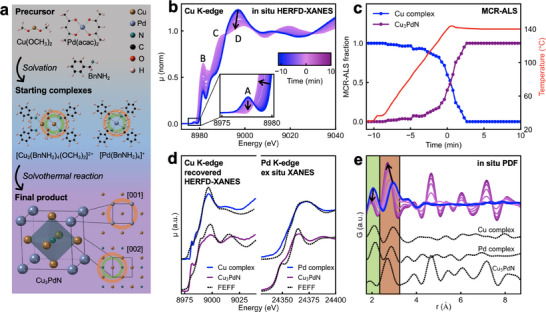
In situ XANES and PDF analysis of the reaction to Cu_3_PdN. a) Schematic of the reaction mechanism. The Cu(OCH_3_)_2_ and Pd(acac)_2_ precursors dissolve in benzylamine to form [Cu_2_(BnNH_2_)_4_(OCH_3_)_2_]^2+^ and [Pd(BnNH_2_)_4_]^+^ complexes, respectively, which form Cu_3_PdN upon heating. The first shell and second shell around the metal atoms are highlighted by green and orange circles, respectively. b) In situ Cu K‐edge HERFD‐XANES data and c) corresponding MCR‐ALS analysis of the in situ Cu K‐edge HERFD‐XANES show the formation of Cu_3_PdN from the Cu starting complex without intermediates. d) XANES spectra of the starting complexes and cation‐site disordered Cu_3_PdN at Cu K‐edge and Pd K‐edge compared to FEFF simulations. The left panel shows the recovered spectra of the Cu starting complex and final product Cu_3_PdN from MCR‐ALS analysis. The right panel shows ex situ XANES spectra of Pd starting complex and Cu_3_PdN. The spectra are in good agreement with the FEFF simulations of the respective structures. e) In situ PDFs G(r). The in situ PDF data is compared to PDF simulations of the Cu and Pd starting complexes and Cu_3_PdN phase in dashed black traces.

The analysis of ex situ PXRD and EXAFS measurements demonstrates the phase purity of the obtained Cu_3_PdN NPs and reveals cation‐side disorder within the anti‐perovskite crystal lattice. To elucidate the reaction pathways leading to the Cu_3_PdN NPs as in **Figure** **4**a, we employ in situ Cu K‐edge HERFD‐XANES and PDF of in situ TS data. Figure 4b shows the in situ Cu K‐edge HERFD‐XANES data. The initial spectrum, blue trace, shows the Cu species in the initial reaction solution at room temperature. The last spectrum, the purple trace, shows the final product Cu_3_PdN after 10 min at 140 °C. The reaction solution is heated up to the reaction temperature of 140 °C with a heating rate of 10 °C min^−1^. The time at which the reaction temperature is reached is defined as 0 min. The room temperature XANES spectrum exhibits a pre‐edge peak at 8977 eV, feature A in Figure 4b, which is the fingerprint of Cu^2+^ originating from the quadrupole‐allowed 1s‐3d transition.^[^ ^63^, ^64^ ^]^ Feature B at the rising edge, at 8983 eV, is due to the 1s to unoccupied 4p transition.^[^ ^63^, ^64^ ^]^ A clear, intense rising edge transition feature is evident for square‐planar coordination.^[^ ^65^, ^66^ ^]^ At elevated temperature, the pre‐edge feature A, disappears, while the white‐line intensity, feature D, decreases, and the edge energy is shifted toward lower energy as indicated by the arrows in Figure 4b. This is a clear indication of the reduction of Cu^2+^ to Cu^1+^. Further, we attribute the simultaneous increase in the intensity of feature B to a linear coordination of Cu^1+^ with two N as present in Cu_3_PdN, which confirms the formation of the nitride phase.^[^ ^65^ ^]^

We apply the multivariate curve resolution by alternating least squares (MCR‐ALS) method to the in situ HERFD‐XANES data and extract the evolution of two distinct Cu components during the reaction, indicating that the initial Cu complex directly reduces to the final Cu_3_PdN phase with no intermediates, as soon as the reaction temperature reaches 140 °C, as shown in Figure 4c. Their corresponding eigenvalue profile is shown in Figure S11 (Supporting Information), while the full‐time series of individual Cu K‐edge HERFD‐XANES spectra, including their MCR‐ALS contributions, can be found in Figure S12 (Supporting Information). By comparing the recovered spectra with FEFF^[^ ^67^ ^]^ simulations, we attribute the starting Cu complex formed when Cu(OCH_3_)_2_ is dissolved in benzylamine (BnNH_2_) to [Cu_2_(BnNH_2_)_4_(OCH_3_)_2_]^2+^, Figure 3d, left panel. In this complex, two Cu ions are bridged and coordinated with two amine molecules and two methoxy groups in a square planar configuration, as visualized in Figure 4a. In addition to FEFF, the structure of the Cu complex is confirmed by EXAFS refinement of the initial spectrum, as shown in Figure S13 (Supporting Information), in which the first coordination around Cu is 2 O atoms at 1.92 Å and 2 N atoms at 1.98 Å. This complex is similar to the one reported already when CuI is dissolved in methanol and BnNH_2_ at room temperature.^[^ ^68^ ^]^ Moreover, we identify the Pd complex present in the reaction solution at room temperature and Cu_3_PdN 10 min after reaching 140 °C by ex‐situ Pd K‐edge XANES in Figure 4d, right panel. When the Pd(acac)_2_ is dissolved in BnNH_2_, the acetylacetonate ligand is released, and the Pd^2+^ ion center is coordinated with four BnNH_2_ molecules in a square‐planar configuration, forming [Pd(BnNH_2_)_4_]^2+^, Figure 4a.^[^ ^69^ ^]^ The Cu K‐edge and Pd K‐edge XANES spectra of the initial precursors and final product, together with their corresponding FEFF simulated spectra, are shown in Figure 4d. The FEFF simulated spectra of Cu_3_PdN are based on a 2 × 2 × 2 supercell (Figure S14, Supporting Information), which allows for introducing partial cation‐site disorder without site occupancies.

We complement the in situ HERFD‐XANES results with in situ PDF analysis as shown in Figure 4e. Consistent with the HERFD‐XANES, PDF analysis shows the prompt formation of Cu_3_PdN NPs upon reaching the reaction temperature of 140 °C, indicated by the emergence of distinct features at high *r* and the shift of the first peak from ≈2.1 to 2.0 Å and the second peak from ≈2.9 to 2.7 Å. The first and second peaks are highlighted in green and orange, respectively, in Figure 3e, and the evolution of the peak position is displayed in Figure S16b (Supporting Information). The first and second shell interatomic distances, corresponding to the first and second peak in the PDF, are consistently highlighted in the representation of the starting complexes [Cu_2_(BnNH_2_)_4_(OCH_3_)_2_]^2+^ and [Pd(BnNH_2_)_4_]^2+^ and the Cu_3_PdN unit cell in Figure 4a. Here we assume the ordered anti‐perovskite structure without cation disorder since the PDF hardly has sensitivity to the cation disorder in a high symmetry structure like Cu_3_PdN, as shown by PDF simulations in Figure S17 (Supporting Information).

The in situ PDF data is compared to PDF simulations of the starting complexes and the Cu_3_PdN phase shown as dashed traces in Figure 4e. The initial PDF, blue trace, matches the superposition of the PDF simulations of the Cu and Pd complex, and the last PDF, purple trace, matches the PDF simulation of phase‐pure Cu_3_PdN as shown in Figure 4e. In Figure S16c (Supporting Information), we analyze the in situ PDF by a linear combination of the initial PDF and the final PDF, which shows consistent reaction pathways to the MCR‐ALS findings of Cu K‐edge HERFD‐XANES. Figure S18 (Supporting Information) shows the full time‐series of the individual PDFs. A detailed description of the analysis procedures and data processing routines is given in the supporting information.

For reaction times exceeding 15 min at 140 °C, in situ time‐resolved PDF refinements (Figures S19–S21, Supporting Information) reveal a further reduction of Cu_3_PdN to the bimetallic Cu_3_Pd phase, which completes within 40 min. The phase transformation is indicated by a peak shift to lower *r*, corresponding to a decrease in the lattice constant as nitrogen atoms leave the crystal lattice. In this context, our ex situ PXRD and EXAFS characterization, combined with in situ XANES and PDF analysis, provides a comprehensive view of the reaction pathway during synthesis, highlighting that accurate control of the reaction parameters is needed to achieve phase‐pure Cu_3_PdN NPs.

**Figure 5:**
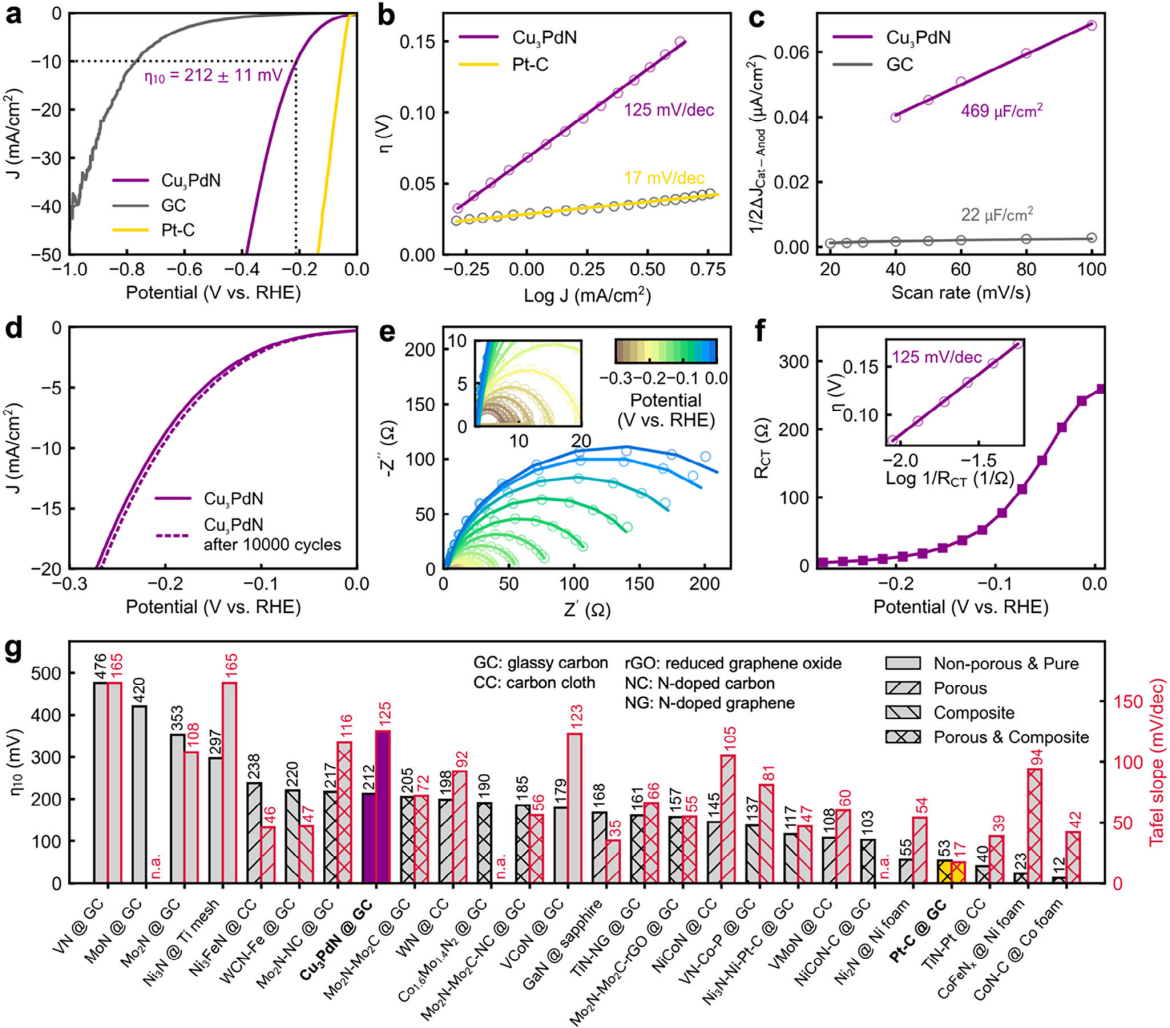
Electrocatalytic performance of Cu_3_PdN NPs for the HER. a) Typical LSV curve of Cu_3_PdN NPs deposited on glassy carbon, compared with Pt‐C and bare glassy carbon electrode, measured at a scan rate of 10 mV s^−1^. b) Tafel plot obtained from the LSV curves. c) double‐layer capacitances obtained from the half‐slope of the capacitive currents extracted from cyclic voltammetry measurements at increasing scan rates. d) LSV scans before and after 10 000 LSV cycles to assess the stability of the electrode. e) Nyquist plot obtained via EIS measurements. f) Dependence of the charge transfer resistance obtained by fitting the EIS data, along with the Tafel plot obtained using the R_CT_ extracted from the impedance fitting (inset). g) Comparison of the obtained overpotential and Tafel slope, highlighted with a colored bar and bold label, with other transition metal nitrides from the literature; missing data points are noted as n.a.: not available.^[^ ^72^, ^73^, ^74^, ^75^, ^76^, ^77^, ^78^, ^79^, ^80^, ^81^, ^82^, ^83^, ^84^, ^85^, ^86^, ^87^, ^88^, ^89^, ^90^, ^91^, ^92^, ^93^, ^94^ ^]^ A detailed overview is given in Table S10 (Supporting Information).

Phase purity is particularly important when evaluating the electrocatalytic performance of a material. For example, Cu_3_PdN has shown superior electrochemical activity for processes such as CO_2_ reduction^[^ ^34^ ^]^ and formic acid oxidation^[^ ^37^ ^]^ compared to the metallic Cu_3_Pd phase. Interestingly, the electrocatalytic performance of Cu_3_PdN NPs for the HER, the cathodic compartment of a water‐splitting cell, has not been reported so far. We therefore deposited our Cu_3_PdN NPs on a glassy carbon electrode and tested their electrochemical performance for the HER in acidic media, using a 0.5 m H_2_SO_4_ aqueous solution as the electrolyte. For comparison, the bare glassy carbon electrode and 20 wt.% Pt/C deposited on glassy carbon with the same catalyst loading was also tested under the same conditions. The linear sweep voltammetry (LSV) scan in **Figure** **5**a reveals an overpotential of η_10_ = 212 ± 11 mV measured at 10 mA cm^−2^ and an onset potential of η_1_ = 67 ± 12 mV defined at 1 mA cm^−2^. The Tafel plot in Figure 5b displays a slope of 125 mV dec^−1^ suggesting that the HER on Cu_3_PdN follows the two‐step Volmer–Heyrovský mechanism:^[^ ^70^ ^]^

(1) $$H_{3}O^{+}+1e^{-}⇌H_{\mathrm{ads}}+H_{2}O\left(\mathrm{Volmer}\mathrm{reaction}\right)$$

(2) $$H_{3}O^{+}+H_{\mathrm{ads}}+1e^{-}⇌H_{2}+H_{2}O\left(\mathrm{Heyrovsk}\overset{´}{y}\mathrm{reaction}\right)$$

where in the first step 1) protons are discharged to form adsorbed hydrogen (H_ads_) followed by the electrochemical desorption of an H_2_ molecule during the second step 2). A Tafel slope close to 120 mV dec^−1^ is often attributed to the Volmer reaction being the rate‐determining step, although recent studies also suggest that such a slope can be obtained for the Heyrovský reaction being the rate‐determining step when the surface of the catalyst is highly covered with adsorbed hydrogen species.^[^ ^71^ ^]^ We also determine the double layer capacitance (C_dl_) of the electrode from cyclic voltammetry measurements as shown in Figure 4c and Figure S21 (Supporting Information), suggesting that the Cu_3_PdN NPs display a high number of active sites and an electrochemically active surface area (ECSA) ≈21 times higher than the bare glassy carbon electrode. We further assess the stability of the Cu_3_PdN NPs during HER operation, as shown in Figure 5d. We performed 10 000 repeated LSV cycles and recorded a small cathodic shift of ≈5 mV after refreshing the electrolyte, suggesting that the Cu_3_PdN NPs show remarkable stability during HER operation. The stability of Cu_3_PdN NPs in the electrolyte and during long‐term electrochemical tests is further confirmed by PXRD and XPS measurements, Figures S24 and S25 (Supporting Information). We further investigate the charge transfer kinetics at the electrode‐electrolyte interface via electrochemical impedance spectroscopy (EIS). In Figure 5e we show a typical Nyquist plot, displaying the presence of a single semicircle with decreasing diameter moving to more cathodic potentials, which suggests that the process is dominated by a charge transfer resistance with the absence of additional processes such as diffusion and mass transport limitations. We model the data with a simple equivalent circuit comprising a resistor (Rs) in series with a parallel combination of a charge transfer resistance (R_CT_) and a constant phase element, as shown in Figure S23 (Supporting Information). Figure 5f shows the dependence of the obtained R_CT_ as a function of the applied potential. At the overpotential of η_10_ = 212 mV, R_CT_ drops to 9.5 Ω, indicating an efficient charge transfer resistance at the catalyst‐electrolyte interface. We further obtain a Tafel plot utilizing the R_CT_ extracted from the EIS fitting (inset in Figure 5f), revealing a slope of 125 mV dec^−1^, which is identical to the one obtained with the voltammetric analysis. This indicates that the HER at the surface of Cu_3_PdN proceeds under pure charge transfer kinetics, without limitations arising from mass transfer diffusion.

Figure 5g presents a comparative overview of the overpotentials and Tafel slopes reported in the literature for various nitrides during HER. The activity of the Cu_3_PdN NPs obtained in this study compares favorably with that of other reported metal nitrides, as the overpotential of most tested nitrides falls within the range of 100–250 mV. It is noteworthy that many of the reported nitrides are either supported on a porous substrate (e.g., Ni_2_N on nickel foam^[^ ^72^ ^]^) or are synthesized as composites (e.g., Mo_2_N‐Mo_2_C^[^ ^73^, ^74^, ^75^ ^]^). These strategies effectively lower the overpotential required for the HER, by utilizing a catalyst support with a very large surface area or by leveraging the synergistic catalytic properties of composite heterostructures. Adopting these approaches could further enhance the performance of Cu_3_PdN in future studies. Nevertheless, our findings indicate that phase‐pure Cu_3_PdN NPs are a promising electrocatalyst for the HER in acidic media.

## 3 Conclusion

This study presents the one‐pot synthesis of phase‐pure, highly crystalline Cu_3_PdN NPs with an average size of 3 nm using benzylamine as a solvent at 140 °C, which is, so far, the lowest reported reaction temperature for Cu_3_PdN synthesis. The Rietveld refinement of the ex situ PXRD patterns demonstrates the phase purity of the obtained Cu_3_PdN NPs, shedding light on the occurrence of Cu_3_Pd impurities often overlooked in previous reports. Crucially, the double‐edge EXAFS analysis reveals the occurrence of disorder in the anti‐perovskite crystal lattice, with ≈25% of the Pd atoms interchanging their position with adjacent Cu atoms, generating a disordered structure with partial cation‐site occupancy. The significantly smaller size of the synthesized Cu_3_PdN NPs, less than half of the previously reported colloidal Cu_3_PdN, provides a high surface area, making them highly suitable for application in electrocatalysis. We showcase that Cu_3_PdN is suitable for the HER, revealing remarkable stability and an overpotential of 212 ± 11 mV at 10 mA cm^−2^, which is comparable to many other reported nitride catalysts. Supporting Cu_3_PdN on porous substrates with a large surface area, such as Ni foam, could be a strategy to further lower the overpotential required for the HER. Furthermore, additional in situ/operando investigations are needed to unveil the HER mechanism at the surface of Cu_3_PdN NPs.

We further monitor the reaction pathway leading to the formation of Cu_3_PdN NPs in benzylamine, employing in situ HERFD‐XANES and PDF analysis of in situ TS data. We observe that the solvation of the Cu and Pd precursors leads to the formation of [Cu_2_(BnNH_2_)_4_(OCH_3_)_2_]^2+^ and [Pd(BnNH_2_)_4_]^2+^ as starting complexes, which then directly react and convert to Cu_3_PdN immediately after reaching the reaction temperature of 140 °C. For an extended reaction time (>15 min), further reduction of Cu_3_PdN to bimetallic Cu_3_Pd occurs, emphasizing that meticulous control of the reaction parameters can prevent the formation of impurities.

In conclusion, our study offers comprehensive insights into the structural and mechanistic aspects of Cu_3_PdN NP formation. The ability to control the cation‐site disorder through parameters such as heating rate, reaction temperature, and time or precursor concentrations could enable to tailor of properties like the band gap, electronic conductivity, and electrocatalytic activity. Additionally, tuning those reaction parameters could impact the size and size distribution of the Cu_3_PdN NPs. The methodologies and findings presented here can be applied to other TMNs, opening avenues for optimizing their properties and expanding their potential applications in energy conversion and beyond.

## 4 Experimental Section

### Chemicals

All Chemicals were purchased from commercial sources and used without further purification: Cu(OCH_3_)_2_ (Thermo Scientific, 98%), Pd(acac)_2_ (Sigma Aldrich, 99%), benzylamine (Sigma–Aldrich, 99%), hexane (Sigma–Aldrich, 99%), N‐methyl‐2‐pyrrolidon (VWR, 99.8%), H_2_SO_4_ (Carl Roth, 96%), methanol (VWR, 99.8%), NAFION (Ion‐Power, 5% in methanol). All chemicals used for the syntheses were stored and handled in the glove box under inert atmosphere (Ar 6.0, c(H_2_O) > 0.1 ppm, c(O_2_) > 0.1 ppm).

### Synthesis

In a typical synthesis, a stock solution was prepared in the glove box by adding Cu(OCH_3_)_2_ (37.7 mg, 0.3 mmol) and Pd(acac)_2_ (30.5 mg, 0.1 mmol) to 5 mL of benzylamine and stirring until all precursors were dissolved. Subsequently, a quantity of 2.5 mL was transferred to the inlet of the heating cell. Instead, for in situ reactions, a quantity of 0.3 mL was transferred to the respective reaction container for in situ XAS or in situ TS, respectively, as explained below. Consequently, the reaction container was assembled in the heating cell, taken out of the glovebox, and heated to 140 °C with a heating rate of 10 °C min^−1^ under vigorous stirring. After the desired reaction time at 140 °C, the heating cell was cooled to room temperature.

After the synthesis, the NPs were washed 3 times with hexane and centrifuged for 10 min at 10 000 rpm. After the last centrifugation step, the nanocrystals were dried under nitrogen flow.

The NP dispersion for HRTEM, STEM:EDX, STEM, and UV–vis characterization was prepared by redispersing the NPs in N‐methyl‐2‐pyrrolidon after the last centrifugation step and discarding the supernatant before drying under nitrogen flow to prevent agglomeration of the synthesized NPs.

### Heating Cell

The synthesis was carried out in a custom‐made heating cell which enables precise control of the ramp rate, reaction temperature, and reaction time, allows for stirring, and operates under solvothermal conditions. The heating cell consists of a metal body and isolating polyether ether ketone (PEEK) (Bieglo) elements. Four heating elements (Cartridge heaters, 24 V, 50 W, Maxiwatt) and a temperature sensor (Pt1000, Honeywell) were used to control the temperature with a temperature controller (Model 336, Lakeshore Cryotronics) and a power supply (EA‐PS 310060–340, Elektro‐Automatik). For the reaction, the heating cell was mounted on top of a magnetic stirring motor (Cimarec i, Thermo Scientific). The reaction takes place in a PEEK inlet as a reaction container. The heating cell was also used for in situ XAS and in situ TS experiments. In situ XAS experiments take place in a PEEK inlet with 0.2 mm wall thickness in reflection geometry. In situ TS experiments take place in a fused silica inlet with 0.5 mm wall thickness in transmission geometry. The heating cell was discussed in detail in the literature.^[^ ^47^ ^]^

It was noted that the synthesis was also feasible in a conventional autoclave. The reaction requires vigorous stirring in addition to an inert atmosphere, which could be achieved by placing the reaction solution and a stirring magnet in the autoclave and heating the autoclave in an oil bath on a magnetic stirring plate. However, precise control over the reaction time was not possible in the conventional autoclave.

### In Situ High Energy Resolution Fluorescence Detected X‐ray Absorption Near Edge Structure (HERFD‐XANES)

In situ Cu K‐edge HERFD‐XANES measurements were performed at ID26^[^ ^95^ ^]^ and ID24^[^ ^96^ ^]^ beamlines of the European Synchrotron Radiation Facility (ESRF), Grenoble, France. The incident X‐ray energy was chosen using the reflection from the Si(111) double crystal monochromator (DCM) and varied from 8970 to 9060 eV at ID26 (radius of curvature R = 1m) and 8970–9040 eV at ID24‐DCM (R = 0.5 m). The spectra were acquired in HERFD mode, using a 5‐analyzer crystal spectrometer in Johann geometry. To detect the maximum intensity of the Cu Kα_1_ emission line (8046 eV), we used five spherically bent Si(444) crystals^[^ ^97^ ^]^ aligned at the Bragg angle of 79.4°. The HERFD‐XANES spectra were recorded in a continuous scan mode every 20–25 s in the energy range of 8970–9060 eV with a step size of 0.2 eV. The overall energy resolution was ≈1.70 eV of FWHM at the Cu Kα_1_ emission line. The beam spot size was ≈200*200 µm, and the beam position on the cell was moved after 2–5 scans to avoid sample damage due to the long exposure to X‐rays. The details of the beam damage study can be found in the SI. The ex situ powder samples were measured as pellets diluted with cellulose.

The Pd K‐edge (24350 eV) EXAFS data were acquired in a transmission mode at the P64 beamline^[^ ^98^ ^]^ of the PETRA III, DESY in Hamburg, Germany. Si (111) monochromator was used for scanning the X‐ray energy from 24135‐24060 eV.

The EXAFS of Cu_3_PdN powder samples were measured both at Cu and Pd K‐edges at the BM23 beamline^[^ ^99^ ^]^ of the ESRF, Grenoble, France. Si(311) DCM was used to monochromatize the incident beam. A double flat mirror was used to ensure a harmonic rejection level better than 10^−5^. All the measurements were taken in transmission mode on pressed pellets diluted with cellulose. The samples were placed between the gas‐filled ionization chambers I_0_ and I_1_ and measured together with a metallic reference foil located between I1 and I2. All the spectra were averaged over three scans to improve the quality of the spectra.

A detailed description of the XANES, EXAFS, and MCR‐ALS analysis procedures was given in the Supporting Information.

### In Situ Total Scattering (TS) and Pair Distribution Function (PDF) Analysis

In situ TS data were acquired at beamline P21.1^[^ ^100^ ^]^ of PETRA III at Deutsches Elektronen–Synchrotron DESY, Hamburg, Germany. Scattering images were recorded every 1 s at an X‐ray energy of 101.39 keV (λ  =  0.1222 Å) using a 2D X‐ray detector (PerkinElmer XRD1621, Varex Imaging Corp.) with 2048 × 2048 pixels and a pixel size of 200 × 200 µm^2^ and a sample‐to‐detector distance of 0.604 m, obtained from a calibration with a LaB_6_ powder standard packed into the fused silica inlet of the heating cell.

A detailed description of the PDF analysis procedures was given in the Supporting Information. The code used for data processing of the PDF data was available at https://gitlab.rrz.uni‐hamburg.de/BAS0906/shm_2024_cu3pdn.

### X‐Ray Photoelectron Spectroscopy (XPS)

The XPS system employed in this work was located in the DESY NanoLab ( https://jlsrf.org/index.php/lsf/article/view/140) in Hamburg, Germany. It is equipped with a monochromated Al Kα source (*h*ν = 1486.6 eV) and a Phoibos 150 hemispherical energy analyzer with a base pressure of 1.5 × 10^−10^ mbar. The pristine Cu_3_PdN powder sample was directly deposited on the carbon tape. The sample on the glassy carbon (GC) electrode (1 cm * 1 cm) after the electrochemical test was washed 5 times with deionised water and 3 times with ethanol. After drying, the samples were then loaded into the UHV chamber and measured after evacuation. The details of the deposition of Cu_3_PdN on the GC electrode were given in the section of Electrochemical characterization. The XPS data are analysed using the Casaxps software. The surface contaminated aliphatic carbon C 1s peak at 284.41 eV is used to calibrate the binding energies. The GL30 (Gaussian 70% & Lorentzian 30%) type profile is used as a fitting function after subtracting the Shirley type background.

### Electrochemical Characterization

The working electrode for the electrochemical measurements was prepared by deposition of the NPs onto an L‐shaped glassy carbon electrode with a surface area of 0.78 cm^2^. Specifically, 40 µL of a 2 mg mL^−1^ dispersion of Cu_3_PdN in methanol were drop‐casted for six times onto the glassy carbon electrode. Afterward, a NAFION solution was prepared by mixing 290 µL of methanol with 2 µL of a 5% NAFION dispersion in methanol. 40 µL of the obtained NAFION dispersion was drop‐casted onto the glassy carbon electrode with the Cu_3_PdN nanoparticles for three times. All the electrochemical measurements were conducted in 0.5 m H_2_SO_4_ employing the typical three‐electrode configuration using a graphite rod and an Ag/AgCl (3 m KCl) as counter and reference electrodes, respectively. Prior to the measurement, the electrolyte was degassed by flushing Ar for ≈15 min. The polarization curves were recorded by liner‐sweep voltammetry at a scan rate of either 10 or 1 mV s^−1^ in a potential range of 0–1 V versus RHE. All polarization curves were not corrected for iR drop. The double‐layer capacitances (C_dl_) were estimated by cyclic voltammetry using a previously reported method.^[^ ^101^ ^]^ Cyclic voltammetry scans were carried out in a 60 mV potential window around the open circuit potential, where no Faradaic processes occur, with variable scan rates, from 10 to 100 mV s^−1^. The double‐layer capacitances (C_dl_) were extracted from the half slope of the linearly fitted curves of the capacitive currents (J_anodic_ – J_cathodic_) versus the scan rates. EIS measurements were carried out in a potential window from 0 to −0.35 V versus RHE with a potential step of 20 mV, an AC sinus amplitude of 10 mV (mean square root voltage V_rms_ = 7.07 mV), a frequency range of 100 kHz–0.1 Hz and an equilibration time of 30 s between each potential step. The EIS spectra were fitted using the software EC‐Lab from Biologic. For all the electrochemical measurements, the potentials acquired with the Ag/AgCl reference electrodes were converted to the RHE scale using the equation E (V vs RHE) = E (V vs Ag/AgCl (3 m KCl)) + 0.197 + 0.059 × pH. All electrochemical measurements were recorded using a Biologic SP‐150 potentiostat.

### Powder X‐ray Diffraction (PXRD) & Rietveld Refinement

PXRD patterns were acquired on a Bruker Advanced D8 with Cu K_α_‐radiation of 8.0478 keV (λ  =  1.5406 Å). Rietveld refinements and PXRD simulations were carried out with GSASII.^[^ ^102^ ^]^ Instrumental parameters have been retrieved by refining a LaB_6_ standard.

### High‐Resolution Transmission Electron Microscopy (HRTEM)

HRTEM images were collected using a JEOL JEM‐2200FS (JEOL Ltd.) with an acceleration voltage of 200 kV. The synthesized sample was dispersed in N‐methyl‐2‐pyrrolidon and deposited on a gold grid.

### STEM:EDX

STEM:EDX images were collected using a Regulus 8220 (Hitachi High Technologies Corp.) with an acceleration voltage of 30 kV and the BFSTEM acquisition mode.

### UV–Vis

UV–vis spectra were collected with a Cary 60 UV–vis spectrometer (Agilent Technologies Inc.) in a quartz cuvette. The sample was dispersed in N‐methyl‐2‐pyrrolidon.

### Elemental Analysis

The Cu concentration was determined using Flame Atomic Absorption Spectroscopy (F‐AAS) with a Soolar S Series spectrometer (Thermo Scientific). The Pd concentration was determined using Inductively Coupled Plasma Optical Emission Spectroscopy (ICP‐OES) using a Spectro Arcos spectrometer (SPECTRO Analytical Instruments GmbH). The N concentration was determined using a EuroEA3000 CHNS‐O Analyzer (EuroVektor S.p.A.).

### Statistical Analysis

The details of the data processing, and treatment of every technique used in this work were explained in their respective subsections in the Supporting Information.

## Conflict of Interest

The authors declare no conflict of interest.

## Supporting information



Supporting Information

## Data Availability

The data that support the findings of this study are openly available in [Repository] at [10.25592/uhhfdm.16631], reference number [16631].
