# Supplementary material for: Cation‐Site Disordered Cu3PdN Nanoparticles for Hydrogen Evolution Electrocatalysis
Source: Small. 2025 Jun 20;21(33):2506838. doi: 10.1002/smll.202506838 (PMC12372454; doi:10.1002/smll.202506838)
Supplement: Supplementary file 1 — Supporting Information [file SMLL-21-2506838-s001.pdf]

NANO · MICRO
small

Supporting Information

for *Small*, DOI 10.1002/smll.202506838

# Cation-Site Disordered $Cu_3PdN$ Nanoparticles for Hydrogen Evolution Electrocatalysis

*Sani Y. Harouna-Mayer, Jagadesh Kopula Kesavan*, Francesco Caddeo, Lian Belgardt, Chia-Shuo Hsu, Lars Klemeyer, Lizzi Kipping, Melike Gumus Akcaalan, Tjark R.L. Groene, Andrea Köppen, Heshmat Noei, Olivier Mathon, Ann-Christin Dippel and Dorota Koziej**

**Cation-site Disordered $Cu_3PdN$ Nanoparticles for Hydrogen Evolution Electrocatalysis**

*Sani Y. Harouna-Mayer, Jagadesh Kopula Kesavan, Francesco Caddeo, Lian Belgardt, Chia-Shuo Hsu, Lars Klemeyer, Lizzi Kipping, Melike Gumus Akcaalan, Tjark R.L. Groene, Andrea Koeppen, Heshmat Noei, Olivier Mathon, Ann-Christin Dippel, Dorota Koziej**

[#]S.Y. Harouna-Mayer, J. Kopula Kesavan and F. Caddeo contributed equally.

S.Y. Harouna-Mayer, J. Kopula Kesavan, F. Caddeo, L. Belgardt, C. Hsu, L. Klemeyer, M. Gumus Akcaalan, T.R.L. Groene, D. Koziej
University of Hamburg, Institute for Nanostructure and Solid-State Physics, Center for Hybrid Nanostructures (CHyN), 22761 Hamburg Germany
E-mail: dorota.koziej@uni-hamburg.de

S.Y. Harouna-Mayer, J. Kopula Kesavan, D. Koziej
The Hamburg Center for Ultrafast Imaging, 22761 Hamburg Germany

H. Noei
Center for X-ray and Nano Science CXNS, Deutsches Elektronen-Synchrotron DESY, 22607 Hamburg, Germany

A. Koeppen
University of Hamburg, Department of Chemistry, 20146 Hamburg, Germany

O. Mathon
European Synchrotron Radiation Facility (ESRF), 38043 Grenoble, France

A. Dippel
Deutsches Elektronen-Synchrotron DESY, 22607 Hamburg, Germany

## Table S1: Review of $Cu_3PdN$ solvothermal synthesis routines.

| **Reference** | **Precursor** | **Solvent/ Ligand** | **Temp.** | **Reaction time** | **Morphology/ Composite** | **Particle Size/ Crystallite Size/ Lat. Par.** | **Degassing** |
|---|---|---|---|---|---|---|---|
| **Parvizian et.al. 2022**[1] | $Cu(NO_3)_2 \cdot 3H_2O$ & $Pd(acac)_2$ | Hexadecane & OAM | 240°C | 5 - 60 min | Cubic | 10.2 nm<br>4.5 ± 1.1 nm<br>3.83 Å | 30 min<br>@ 50 °C |
| **Yao et.al. 2023**[2] | $Cu(NO_3)_2 \cdot 3H_2O$ & $Pd(acac)_2$ | 1-Octadecene & OAM | 240°C | 15 min | Spherical/ On activated carbon | 18 nm<br>-<br>- | 10 min<br>@120 °C |
| **Jia et.al. 2021**[3] | $Cu(NO_3)_2 \cdot 3H_2O$ & $Pd(acac)_2$ | 1-Octadecene & OAM | 230°C | 15 min | Spherical/ On rGO | 16 nm<br>4 ± 4.6 nm<br>3.81 Å | 5 min @ 120°C |
| **Lord et.al. 2019**[4] | $Cu(NO_3)_2 \cdot 3H_2O$ & $Pd(acac)_2$ + Metal NP seeds | 1-Octadecene & OAM | 190°C | 30 min | Heterostructures/ Cu3PdN @ Pt or @ Pd | -<br>-<br>- | 60 min @ 120 °C |
| **Jia et.al. 2016**[5] | $Cu(NO_3)_2 \cdot 3H_2O$ & $Pd(acac)_2$ | 1-Octadecene & OAM | 250°C | 30 min | Spherical | 11.3 ± 2.5 nm<br>11.2 nm<br>- | 10 min @ 120 °C |
| **Vaughn et.al. 2014**[6] | $Cu(NO_3)_2 \cdot 3H_2O$ & $Pd(acac)_2$ | 1-Octadecene & OAM | 240°C | 5 - 15 min | Quasi-cubic | 16 ± 2 nm<br>8 nm<br>3.82 Å | 10 min @ 120 °C |
| **Li et.al. 2021**[7] | $Cu(NO_3)_2 \cdot 3H_2O$ & $Pd(acac)_2$ | 1-Octadecene & OAM | 245°C | 20 min | Quasi-cubic | 20 ± 2 nm<br>-<br>- | - |
| **This work** | $Cu(OCH_3)_2$ & $Pd(acac)_2$ | Benzylamine | 140°C | 5 - 15 min | Spherical | 3.5 ± 1.0 nm<br>3.5 ± 1.0 nm<br>3.83 Å | - |

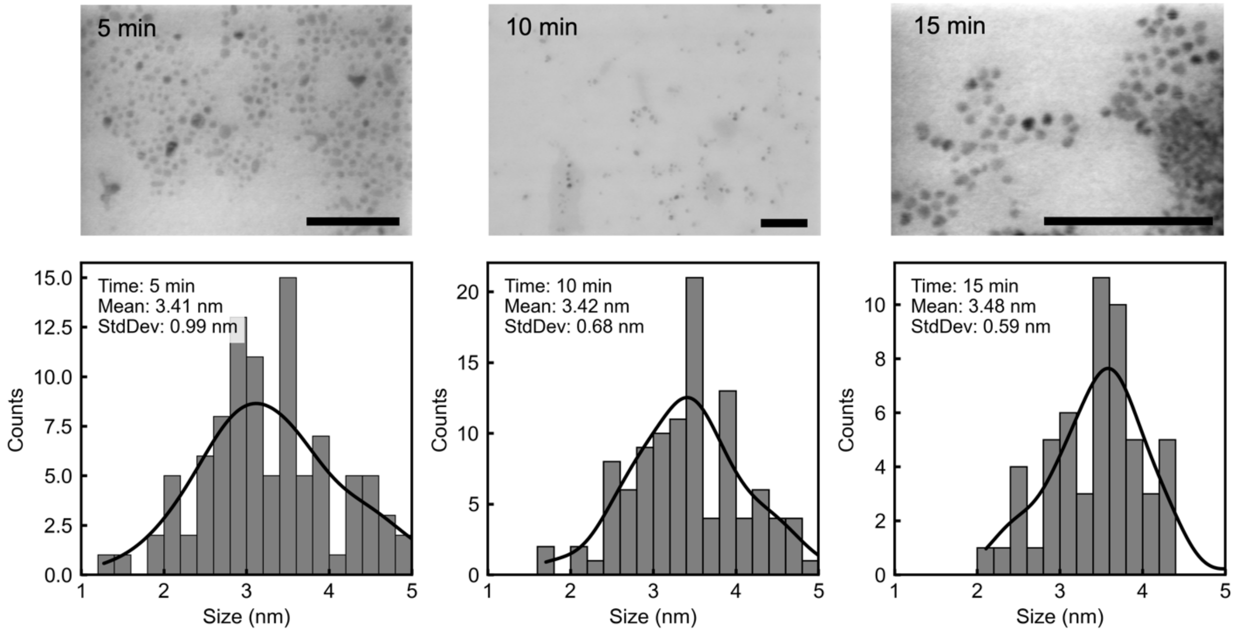


**Figure S1: STEM images and size distribution of $Cu_3PdN$ grown for 5, 10 and 15 min reaction time at 140°C.** Number of measured particles (n): 5 min: 103, 10 min: 107, 15 min: 56. Scalebar 50 nm.

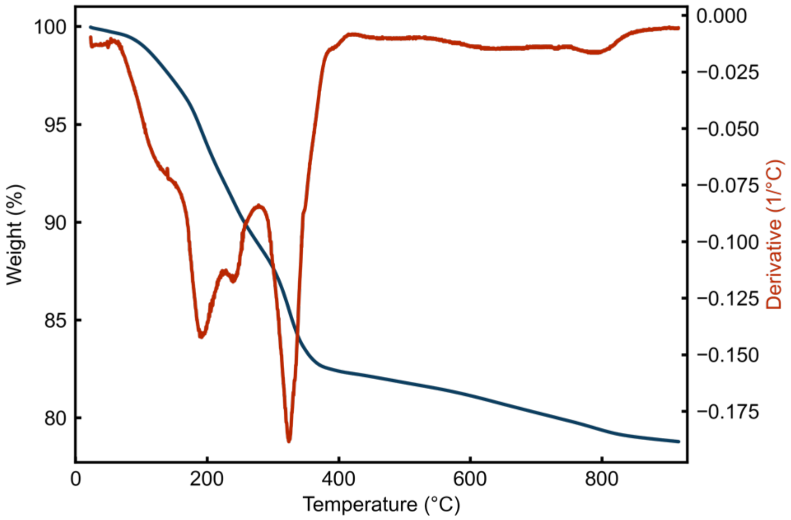


**Figure S2: TGA performed under a nitrogen atmosphere of $Cu_3PdN$ 15 min reaction time at 140°C.**

## Phase Impurity

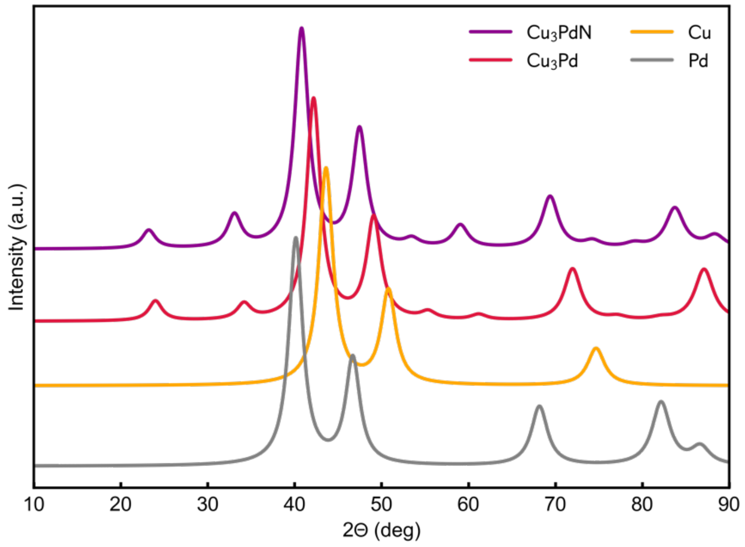


**Figure S3: Simulated PXRD pattern of $Cu_3PdN$, $Cu_3Pd$, Cu and Pd.** All patterns are simulated with GSASII[8] using Cu $K_\alpha$-radiation ($\lambda = 1.5406$ Å) and a crystallite size of 5 nm.

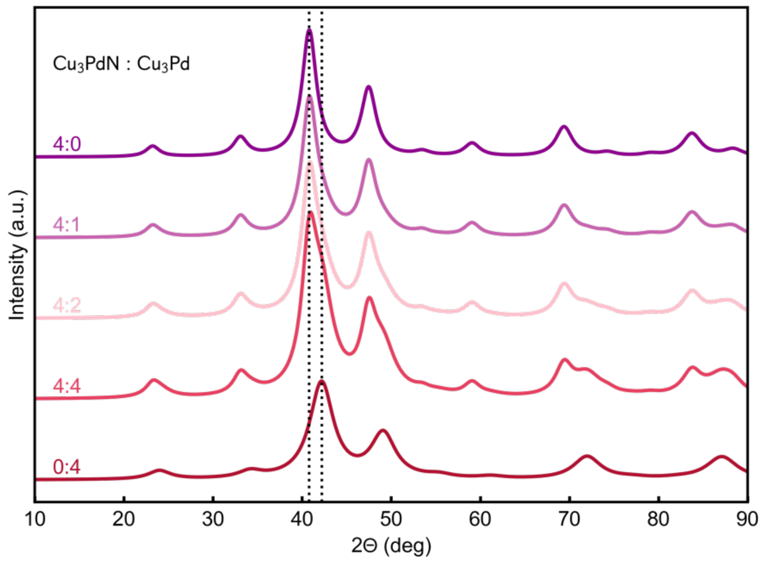


**Figure S4: Simulated two-phase PXRD pattern of $Cu_3PdN$ and $Cu_3Pd$ at varying phase ratios.** All patterns are simulated with GSASII[8] using Cu $K_\alpha$-radiation ($\lambda = 1.5406$ Å). $Cu_3PdN$ and $Cu_3Pd$ phases are simulated with a crystallite size of 5 and 3 nm, respectively. The simulated

two-phase PXRD patterns comprise individual computed patterns of the two phases with the respective crystallite size which are added with the respective phase ratios.

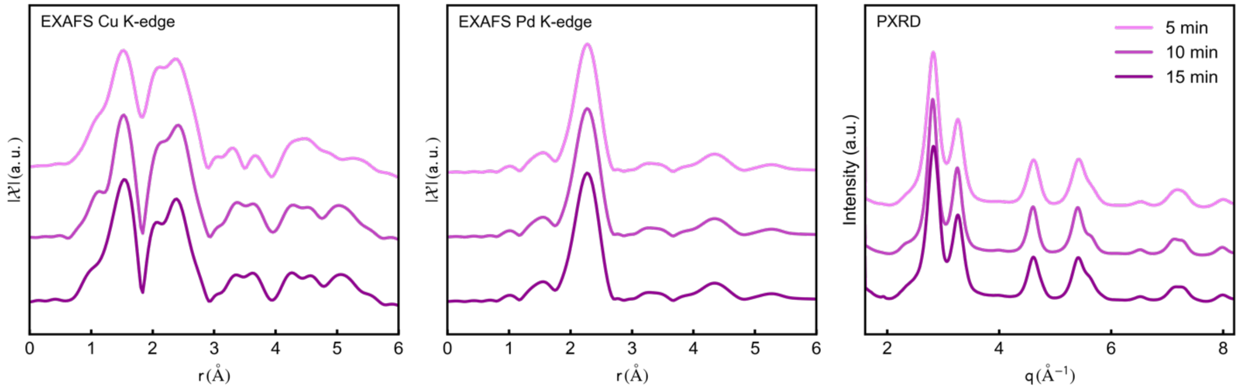


**Figure S5: Waterfall plot of EXAFS Cu K-edge, EXAFS Pd K-edge and PXRD of $Cu_3PdN$ grown for 5, 10 and 15 min reaction time at 140°C.**

**EXAFS refinements**

The cation-site disorder in $Cu_3PdN$ nanoparticles is determined by EXAFS refinement on ordered and disordered structural models, as shown in Figure S6. Below we describe the refinement procedures for the ordered and disordered models. More details on the EXAFS data processing and the refinement procedure are available in the section **Technical information on XANES and EXFAS analysis**.

$Cu_3PdN$ exhibits the anti-perovskite structure which consists of corner-shared, N-centered Cu-N octahedra with a Pd atom in the central cubic position. In the ordered case, the local coordination around Cu in the first shell is Cu-N at 1.91 Å; the second shell is composed of Cu-Cu and Cu-Pd coordination at 2.70 Å and the third shell is Cu-Cu coordination at 3.86 Å. In the case of Pd, the first coordination is Pd-Cu at 2.70 Å, second coordination is Pd-N at 3.25 Å and the third shell coordination is Pd-Pd at 3.86 Å. Based on these components including the multiple scattering components, the Cu and Pd K-edge data were fitted in the case of ordered structure and fits are shown in Figure S6.

In the case of disordered structure, two crystallographic models are considered. They are (i) ordered anti-perovskite structure: as explained above and (ii) fully disordered anti-perovskite structure: Pd and Cu are fully displaced their positions. The scattering components of both structures are used as linear combination to fit the data. i.e. $S_0^2 * x$ and $S_0^2 * (1 - x)$ and x is considered as a refining parameter. Obviously, the Cu – N, Pd – N, Cu – Cu and Pd – Pd scattering components are not common to both edges. The Debye-Waller (DW) factors for multiple scattering components are fixed to twice the single scattering ones which is necessary to limit the free parameters for the fit. The DW factors for some of the components in the second and third coordination shell are high due to the cation disorder. It is also seen in figure S7 that the intensity of the peaks in the simulated spectra is high compared to the experimental data. The scattering components used for the fit and their corresponding distance variation parameter and DW factors with graphical representations are listed in Table S2. The refinement results of all the three samples are shown in Table S3 and the spectra with best fits in R-space and in k-space are shown in Figure S6.

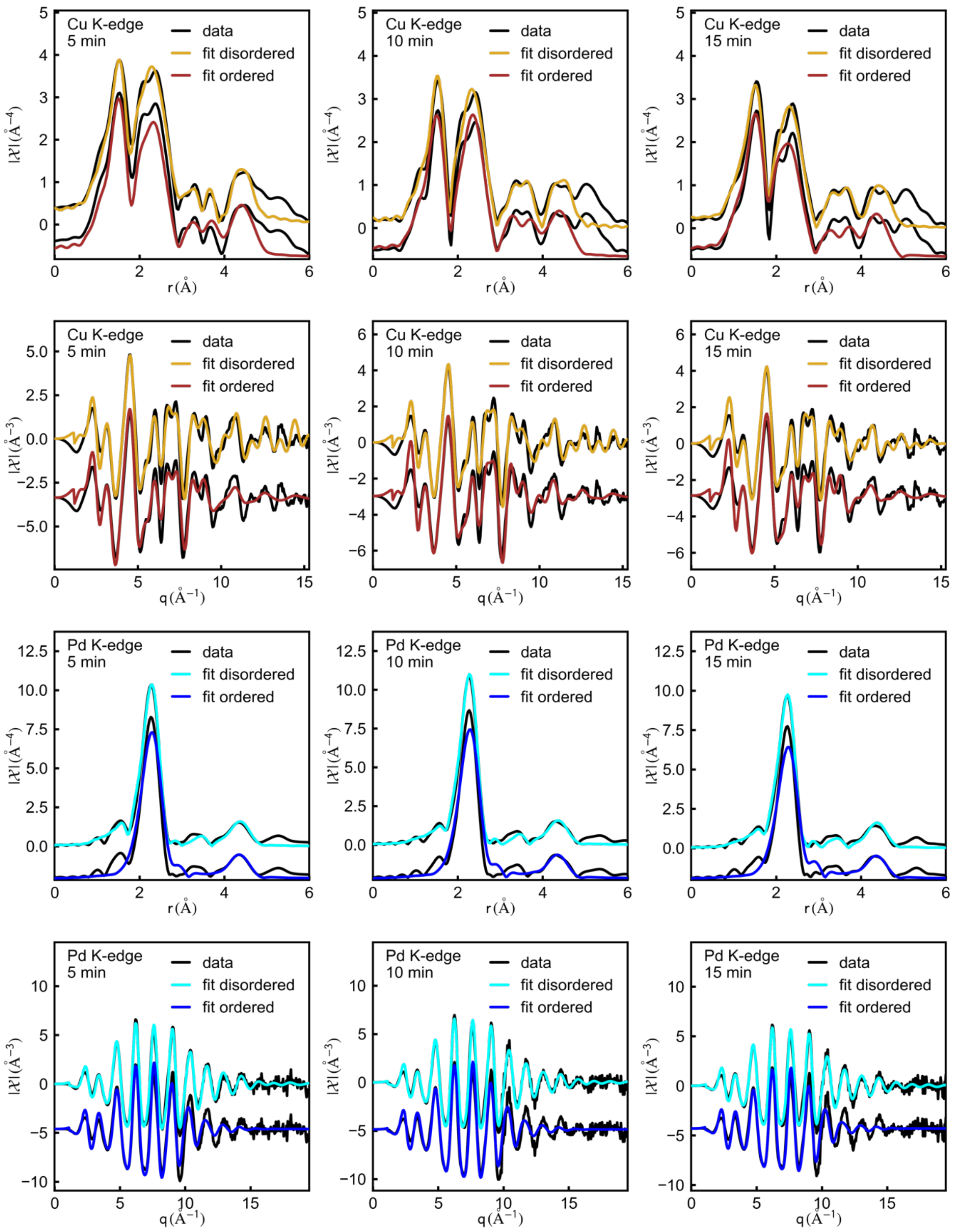


**Figure S6: Double-edge Cu K-edge and Pd K-edge EXAFS fit of $Cu_3PdN$ using the disordered and ordered anti-perovskite structure of $Cu_3PdN$ for NPs grown for 5, 10 and 15 min reaction time at 140°C.** Refined parameters are shown in Table S2 and S3.

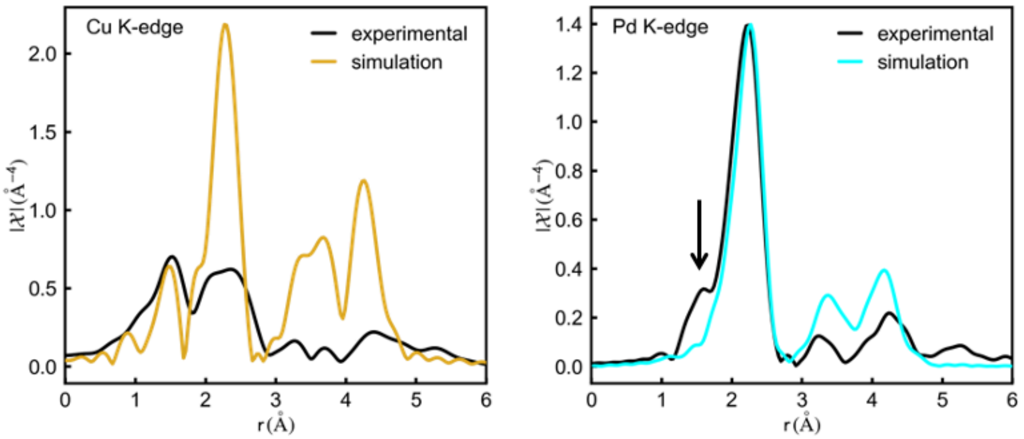


**Figure S7. Comparison of experimental and FEFF-simulated Fourier-transformed Cu K-edge and Pd K-edge EXAFS data of ordered $Cu_3PdN$.** The simulated data is multiplied by a factor of 0.25. A clear peak at 1.6 Å, indicated by an arrow arising from the Pd-N coordination in the Pd K-edge experimental spectrum, emphasises the presence of the cation site disorder. The low intensity of the peaks related to the second and the third coordination in the experimental data, compared to the simulated ones, due to the high DW values, further confirms the disorder in the cation lattice.

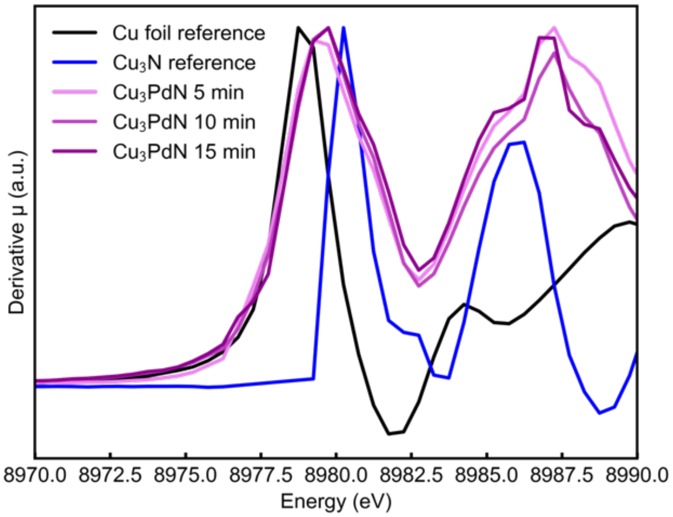


**Figure S8: First derivative XANES spectra of $Cu_3PdN$ grown for 5, 10 and 15 min reaction time at 140°C compared with the reference $Cu_3N$ and Cu foil.**

**Table S2. The scattering paths used to fit the corresponding Cu and Pd K-edge EXAFS data in the case of the disordered structure.** The graphical description is Cu: orange; Pd: cyan; N: green.

| No | Scattering Path (legs) | Edge | Degeneracy (n) | $\frac{1}{2}R_{path}$ (Å) | Distance variation parameter | Debye Waller factor | Graphical description |
|---|---|---|---|---|---|---|---|
| 1 | Cu – N1 – Cu | Cu | 2 | 1.91 | $\Delta r_1$ | $\sigma^2_{Cu1}$ | 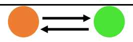 |
| 2 | Pd – N1– Pd | Pd | 2 | 2.00 | $\Delta r_2$ | $\sigma^2_{Pd1}$ | 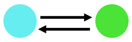 |
| 3 | Cu – Cu 1– Cu | Cu | 12 | 2.69 | $\Delta r_3$ | $\sigma^2_{Cu2}$ | 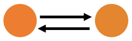 |
| 4 | Cu – Pd 1– Cu | Cu & Pd | 8 | 2.71 | $\Delta r_4$ | $\sigma^2_{Cu3}$ | 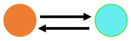 |
| 5 | Cu – Pd 1– Cu | Cu & Pd | 4 | 2.63 | $\Delta r_5$ | $\sigma^2_{Cu4}$ | 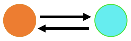 |
| 6 | Pd – Pd1 – Pd | Pd | 4 | 2.73 | $\Delta r_6$ | $\sigma^2_{Pd2}$ | 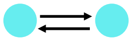 |
| 7 | Pd – N2 – Pd | Pd | 8 | 3.34 | $\sqrt{3}.\Delta r_2$ | $\sigma^2_{Pd3}$ | 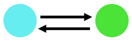 |
| 8 | Pd – Pd 2– Pd | Pd | 6 | 3.83 | $\sqrt{2}.\Delta r_4$ | $\sigma^2_{Pd4}$ | 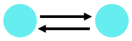 |
| 9 | Cu – N1 – Cu2 - Cu | Cu & Pd | 4 | 3.83 | $\sqrt{2}.\Delta r_4$ | $2.\sigma^2_{Pd4}$ | 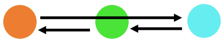 |
| 10 | Cu – N1 – Cu2 – N1 – Cu | Cu & Pd | 2 | 3.83 | $\sqrt{2}.\Delta r_4$ | $2.\sigma^2_{Pd4}$ | 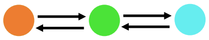 |
| 11 | Pd – N 1– Cu1 - Pd | Pd | 48 | *3.97* | $2.\Delta r_2$ | $2.\sigma^2_{Pd1}$ | 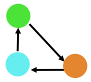 |
| 12 | Pd – Cu2 – Pd | Cu & Pd | 24 | *4.70* | $\sqrt{3}.\Delta r_4$ | $\sigma^2_{Cu5}$ | 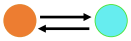 |
| 13 | Cu – Cu3 – Cu | Cu | 16 | *4.70* | $\sqrt{3}.\Delta r_3$ | $\sigma^2_{Cu6}$ | 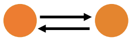 |

**Table S3. EXAFS refined parameters.** The error is given by the standard deviation of the refined parameter and shown in the parentheses. *Cu K $E_0$* is calculated from the maximum of the first derivative of the corresponding XANES spectra.

| Sample/Results | 5 min | 10 min | 15 min |
|---|---|---|---|
| **Cu K $E_0$ (eV)** | 8979.60 | 8979.59 | 8979.65 |
| **x (%)** | 69.3 (3.9) | 76.5 (4.3) | 75.7 (4.4) |
| **$R_{Cu-N1}$ (Å)** | 1.908 (7) | 1.909 (6) | 1.917 (2) |
| **$\sigma^2_{Cu-N1}$** | 0.0039 (6) | 0.0048 (5) | 0.0046 (5) |
| **$R_{Pd-N1}$ (Å)** | 2.001 (6) | 1.999 (9) | 2.004 (8) |
| **$\sigma^2_{Pd-N1}$** | 0.0035 (17) | 0.0038 (25) | 0.0040 (78) |
| **$R_{Cu-Pd1}$ (Å)** | 2.641 (7) | 2.630 (7) | 2.635 (6) |
| **$\sigma^2_{Cu-Pd1}$** | 0.0050 (6) | 0.0047 (7) | 0.0048 (6) |
| **$R_{Cu-Cu1}$ (Å)** | 2.711 (11) | 2.726 (18) | 2.724 (16) |
| **$\sigma^2_{Cu-Cu1}$** | 0.0242 (10) | 0.0249 (18) | 0.0266 (15) |
| **$R_{Cu-Pd1}$ (Å)** | 2.697 (16) | 2.689 (19) | 2.700 (8) |
| **$\sigma^2_{Cu-Pd1}$** | 0.0098 (7) | 0.0107 (7) | 0.0114 (7) |
| **$R_{Pd-Pd1}$ (Å)** | 2.731 (23) | 2.730 (22) | 2.738 (30) |
| **$\sigma^2_{Pd-Pd1}$** | 0.0092 (8) | 0.0079 (8) | 0.0075 (7) |
| **$R_{Pd-N2}$ (Å)** | 3.240 (71) | 3.262 (55) | 3.300 (17) |
| **$\sigma^2_{Pd-N2}$** | 0.0121 (27) | 0.0132 (24) | 0.0143 (23) |
| **$R_{Cu-Cu2}$ (Å)** | 3.862 (32) | 3.861 (31) | 3.872 (42) |
| **$\sigma^2_{Cu-Cu2}$** | 0.0131 (36) | 0.0113 (31) | 0.0135 (44) |
| **$R_{Cu-Cu3}$ (Å)** | 4.705 (14) | 4.707 (16) | 4.718 (27) |
| **$\sigma^2_{Cu-Cu3}$** | 0.0241 (29) | 0.0248 (25) | 0.0265 (23) |
| **$R_{Pd-Cu2}$ (Å)** | 4.718 (27) | 4.722 (31) | 4.728 (37) |
| **$\sigma^2_{Pd-Cu2}$** | 0.0169 (11) | 0.0179 (11) | 0.0176 (12) |

**XRD and Rietveld Refinement Analysis**

We test the hypothesis of cation disorder with PXRD. First, in Figure S9, we compare simulations for PXRD patterns of the ordered $Cu_3PdN$ structure without atomic displacements (approach A), the $Cu_3PdN$ structure with fully displaced Cu and Pd atoms (approach B), and fully displaced N atoms (approach C) and partially displaced Cu/Pd or N with adjusted atomic site occupancy to match the ratio of the displacement as determined by the EXAFS fit. We note that the structure of displaced Cu/Pd and displaced N, approach B and C, respectively, is identical in the first coordination shell but varies beyond. The simulated patterns exhibit little changes in relative peak intensity, most notably for peak (100) at $q$ = 1.6 $Å^{-1}$ and (110) at $q$ = 2.3 $Å^{-1}$, corresponding to $2\Theta$ = 23° and $2\Theta$ = 33°, respectively, considering Cu K-alpha radiation. The peak intensity (100) and (110) notably decreases in the case of the displaced Cu and Pd model. Our PXRD pattern and literature reports display a similar pattern.[1–7] However, the peak intensities in PXRD patterns are influenced by atomic displacement parameters (ADP), which are typically included in Rietveld refinement procedures to account for the uncertainty of the atomic position within the crystal lattice due to thermal vibration or static disorder.[9] In Figure S10, we conduct Rietveld refinements using the ideal $Cu_3PdN$ structure and partially displaced N atoms or Cu and Pd atoms. Table S4 lists the refined parameters. The refinements demonstrate good agreement across all models, although the refinement with partially displaced N atoms has unphysical ADP values indicating unlikely N displacement. Hence, with Rietveld refinements alone we cannot differentiate between the ordered $Cu_3PdN$ and the disordered structure, i.e. the displacement of Cu and Pd, but exclude the potential displacement of N atoms.

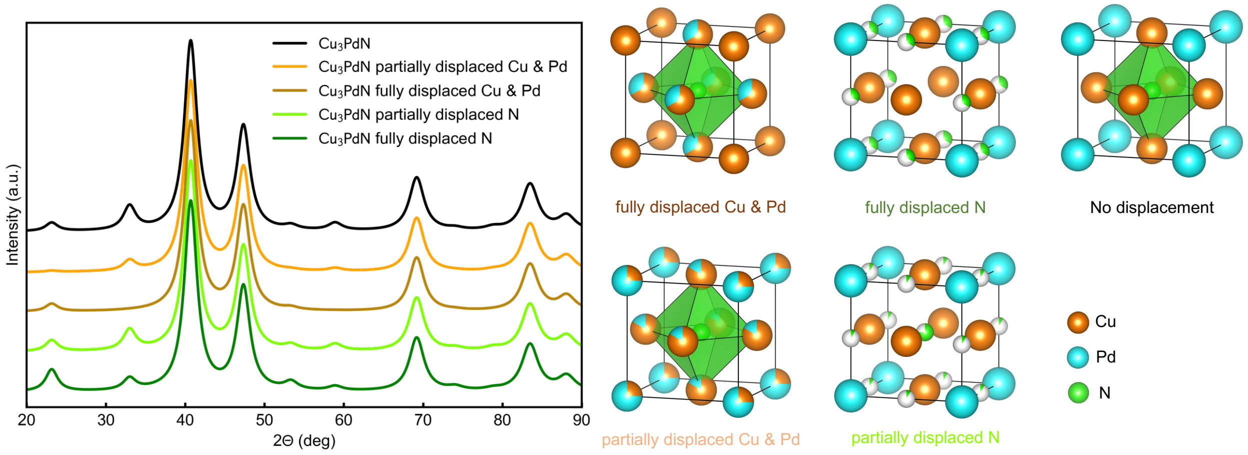


**Figure S9: PXRD simulations of $Cu_3PdN$ with no displacements, partially and fully displaced Cu & Pd or N atoms.**

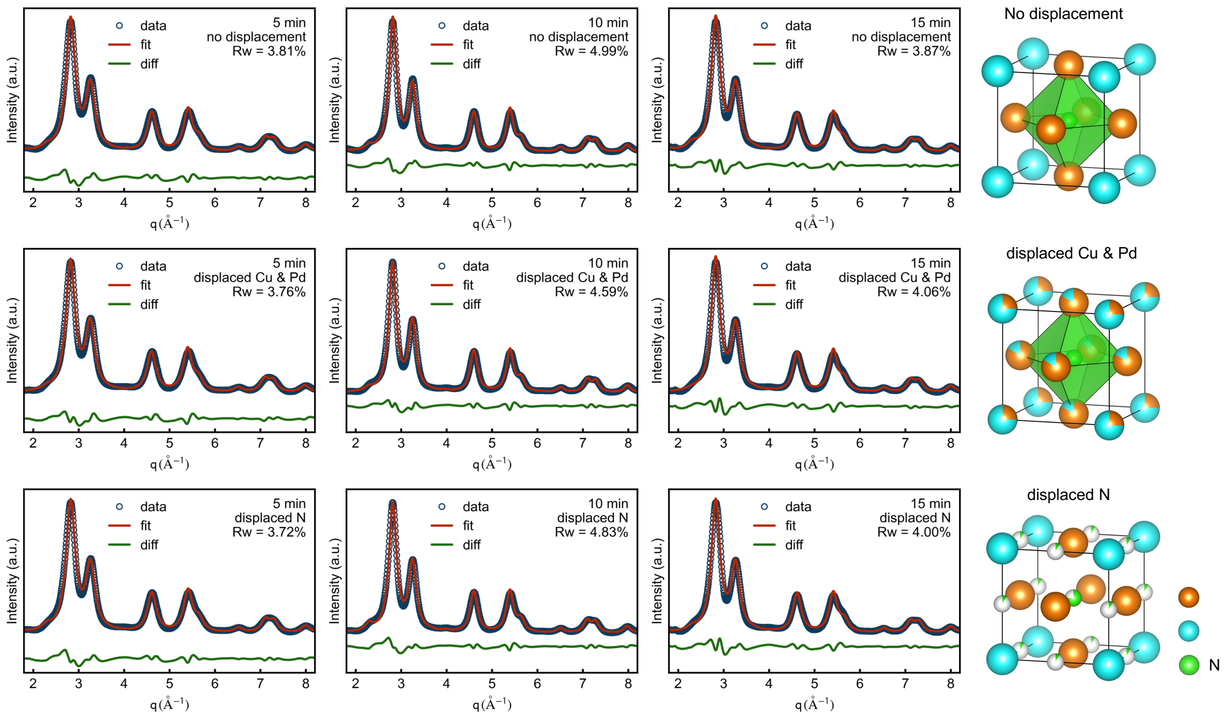


**Figure S10: Rietveld refinements of $Cu_3PdN$ at 5, 10 and 15 min reaction time at 140°C.** The patterns were refined using GSASII[8]. Refined parameters are listed in Table S3.

**Table S4: Retrieved Parameters of Rietveld Refinement Results of $Cu_3PdN$ at 5, 10 and 15 min reaction time at 140°C.** *Displaced atom. The displacement takes place in {a/2, a/2, 0} from the original position of the atom for Cu, Pd and N. Unphysical values are marked in red.

| | 5 min | | | 10 min | | | 15 min | | |
|---|---|---|---|---|---|---|---|---|---|
| **Displacement** | **-** | **Cu/Pd** | **N** | **-** | **Cu/Pd** | **N** | **-** | **Cu/Pd** | **N** |
| **$R_W$ (%)** | 3.813 | 3.757 | 3.716 | 4.986 | 4.587 | 4.825 | 3.873 | 4.063 | 3.995 |
| **$Chi^2$** | 41.67 | 40.54 | 39.62 | 115.3 | 98.54 | 108.9 | 57.33 | 63.29 | 61.15 |
| **a (Å)** | 3.838 | 3.838 | 3.838 | 3.847 | 3.838 | 3.847 | 3.833 | 3.833 | 3.833 |
| **size (nm)** | 2.6 | 2.7 | 2.7 | 3.3 | 3.3 | 3.3 | 2.7 | 2.9 | 2.9 |
| **$U_{iso}$ Cu ($Å^{-2}$)** | 0.0030 | 0.0070 | 0.0038 | 0.0041 | 0.0112 | 0.0083 | 0.0041 | 0.0102 | 0.0059 |
| **$U_{iso}$ Pd* ($Å^{-2}$)** | | 0.0104 | | | 0.0224 | | | 0.0257 | |
| **$U_{iso}$ Pd ($Å^{-2}$)** | 0.0385 | 0.0288 | 0.0393 | 0.0358 | 0.0115 | 0.0269 | 0.0447 | 0.0027 | 0.0411 |
| **$U_{iso}$ Cu* ($Å^{-2}$)** | | 0.0286 | | | 0.0137 | | | 0.0168 | |
| **$U_{iso}$ N ($Å^{-2}$)** | 0.7424 | 0.2763 | 0.2267 | 0.5781 | 0.0780 | 0.0055 | 0.0623 | 0.0712 | 0.7641 |
| **$U_{iso}$ N* ($Å^{-2}$)** | | | 27.10 | | | 1.956 | | | -119.5 |

**Technical information on XANES and EXAFS analysis**

The XANES and EXAFS data shown in this report have been acquired at different beamlines as listed in Table S5.

**Table S5: Summary of the beamlines and synchrotrons utilised for collecting XAS data, as referenced in the figures of this report.**

| | **Method** | **Beamline, Synchrotron** |
|---|---|---|
| Figure 1 | Cu K-edge HERFD-XANES | ID24, ESRF |
| Figure 2 | Cu & Pd K-edge EXAFS | BM23, ESRF |
| Figure 3 | Cu K-edge HERFD-XANES | ID24, ESRF |
| | Pd K-edge XANES | P64, DESY |
| Figure S5 | Cu & Pd K-edge EXAFS | BM23, ESRF |
| Figure S6 | Cu & Pd K-edge EXAFS | BM23, ESRF |
| Figure S7 | Cu & Pd K-edge EXAFS | BM23, ESRF |
| Figure S8 | Cu K-edge XANES | BM23, ESRF |
| Figure S12 | Cu K-edge HERFD-XANES | ID24, ESRF |
| Figure S13 | Cu K-edge EXAFS | P64, DESY |
| Figure S14 | Cu K-edge HERFD-XANES | ID26, ESRF |

In situ HERFD-XANES data were preliminarily viewed using the PyMCA software package[10] and processed using a self-written Python code based on available packages. The data was imported using Silx[11], treated with a Whittaker filter[12] and further processed with the NumPy[13] and SciPy[14] packages. Ex situ powder samples XANES data were pre-treated using the Athena[15] of the Demeter software package.

The pretreatment process for all EXAFS spectra was performed using the Athena program of the Demeter IFEFFIT software package, and the data were analyzed Artemis code of the Demeter software package[15] using simulated scattering paths calculated by FEFF 6.0. The Fourier transform of the EXAFS spectra were obtained in the range of 3 – 13.5 Å$^{-1}$ and 3 - 18.5 Å$^{-1}$ with $k^3$ weight for both Cu K and Pd K-edge data, respectively. The data were fitted in R space in the range of 1 – 4.7 Å. The many-body amplitude reduction factor ($S_0^2(Cu) = 0.955, S_0^2(Pd) =$

0.935 ) and the common energy origin shift ($\Delta E_0$(Cu) = 5.05, $\Delta E_0$(Cu) = 4.68) were fixed to the values obtained from a fit of the Cu and Pd foils spectra, respectively. The fitting parameters were the interatomic distances (R), the Debye-Waller factors ($\sigma^2$), and x. The goodness of the fit (R) was below 0.01 for all the spectra.

The Multivariate Curve Resolution by Alternating Least Squares (MCR-ALS)[16] method is briefly reviewed, followed by detailed fit results. MCR-ALS is an emerging data analysis technique used to extract the reaction pathway from in situ XANES data. It allows for modelling an experimental dataset D (including q spectra), as the product of an S matrix, composed of N (with N < q) pure spectra and a matrix C, whose elements correspond to signal-related concentration profiles.

$$D = CS^T + E \qquad (1)$$

Here, in the case of time-resolved XANES spectra, D is the experimental data with each row corresponding to a measured spectrum, rows of $S^T$ are spectra of uncorrelated variables and columns of C are the concentration profiles of each component over time. E represents the error matrix associated to the reconstruction. For this MCR-ALS analysis we used the Graphical User Interface (GUI) by Jaumot and co-workers which is freely available using MATLAB R2011b.[17,18] After guessing of initial C and $S^T$, which we do by means of the purest variables detection method so called SIMPLISMA algorithm[19] with allowed noise parameter is fixed to 3 %, a set of linear equations is iteratively solved, alternatingly keeping C or ST constant until the change in the standard deviation of E falls below a certain convergence criterion. For MCR-ALS, the data were analyzed in the energy range of 8970-9060 eV for Cu K-edge and 24280-24450 eV for Pd K-edge data sets. The optimization routine successfully converged after 50 iterations, resulting in the final ALS quality control parameters such as lack of fit, $R^2$ and $\sigma$. The quality estimates of a converged fit arise from the unexplained residuals E, or the difference between the experimental data and the model, with a lack of fit is given by

$$Lack\ of\ fit\ (\%) = 100\sqrt{\frac{\Sigma_{i,j} e_{ij}^2}{\Sigma_{i,j} d_{ij}^2}} \qquad (2)$$

Where $d_{ij}$ is a data matrix element and $e_{ij}$ is the corresponding element of the residual matrix E. Additionally, the variance explained in the model can be estimated from

$$R^2 = \frac{\Sigma_{i,j}\, d_{ij}^2 - \Sigma_{ij} e_{ij}^2}{\Sigma_{ij} d_{ij}^2} \quad (3)$$

and the standard deviation of the residuals is given by

$$\sigma = \frac{\Sigma_{i_1 j} e_{ij}^2}{nm} \quad (4)$$

Where n, m refers to the dimensions of D.

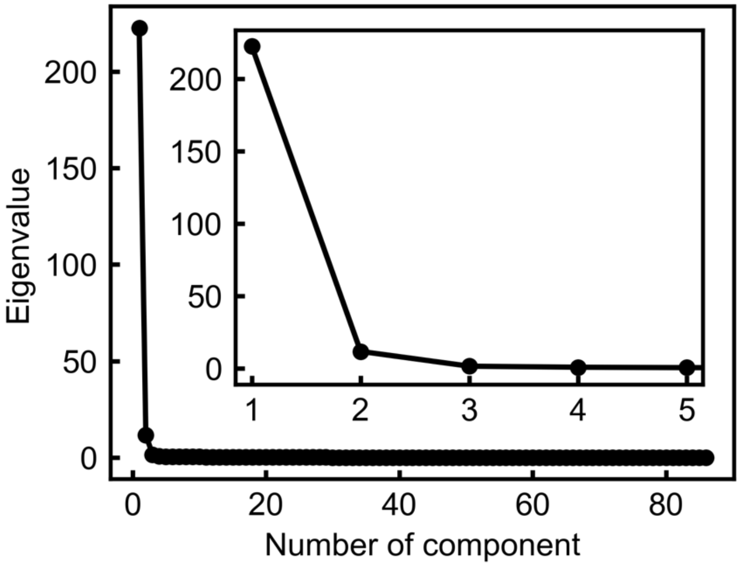


**Figure S11: Eigen value profile of MCR-ALS analysis.**

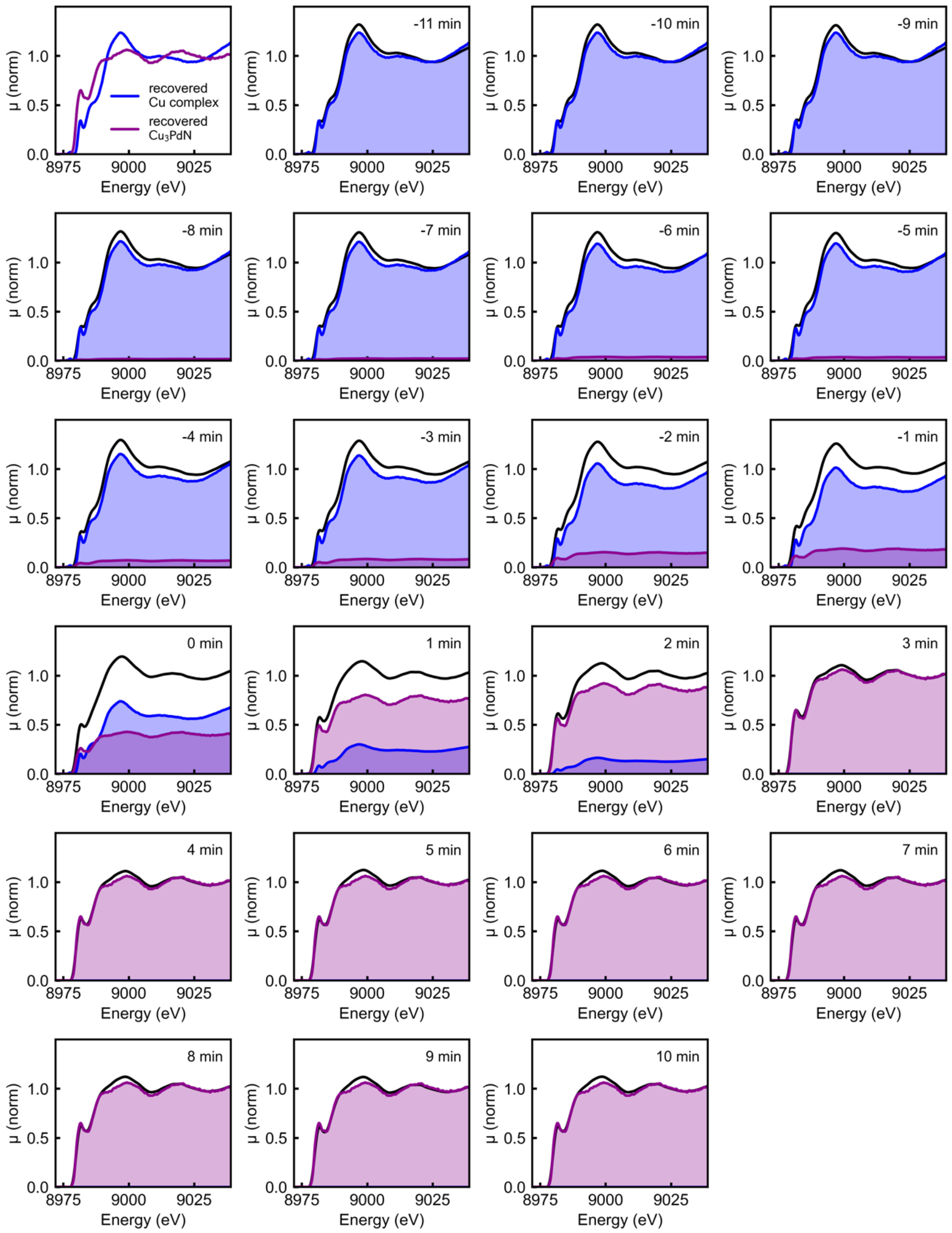


**Figure S12: Time series of Cu K-edge HERFED-XANES spectra compared with contributions from MCR-ALS recovered spectra which are shown in Figure 3c,d.** The black curve shows the measured spectra. The blue and violet curve show the MCR-ALS recovered contribution of the Cu complex and $Cu_3PdN$, respectively.

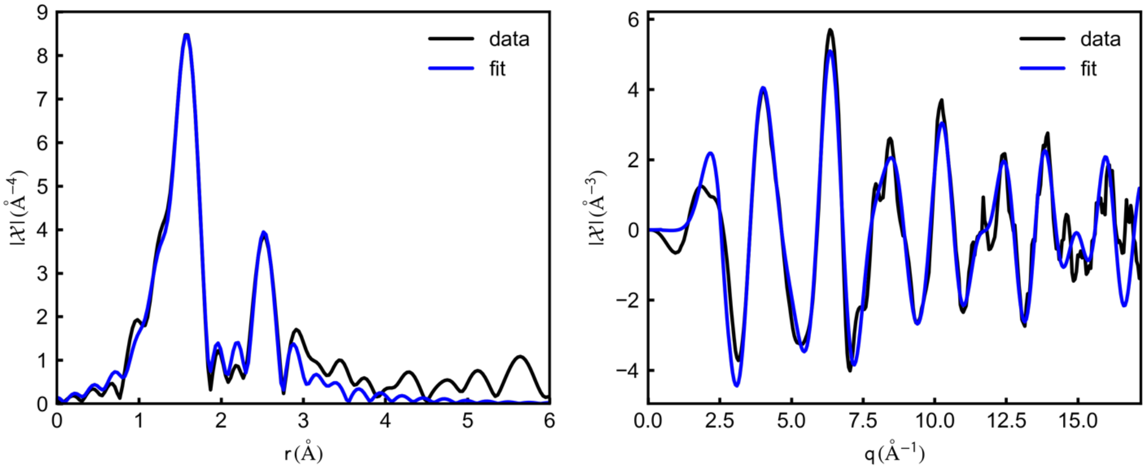


**Figure S13. Fourier transformed Cu K-edge EXAFS data of the initial complex fitted using the complex shown in Figure 3.** The fit reveals that the first coordination shell is composed of 2 N atoms and 2 O atoms at 1.92 Å and at 1.98 Å, respectively. The second shell is composed of 4 C atoms and 1 Cu atom at 2.92 Å and at 3.04 Å, respectively which clearly corresponds to the complex shown in Figure 3.

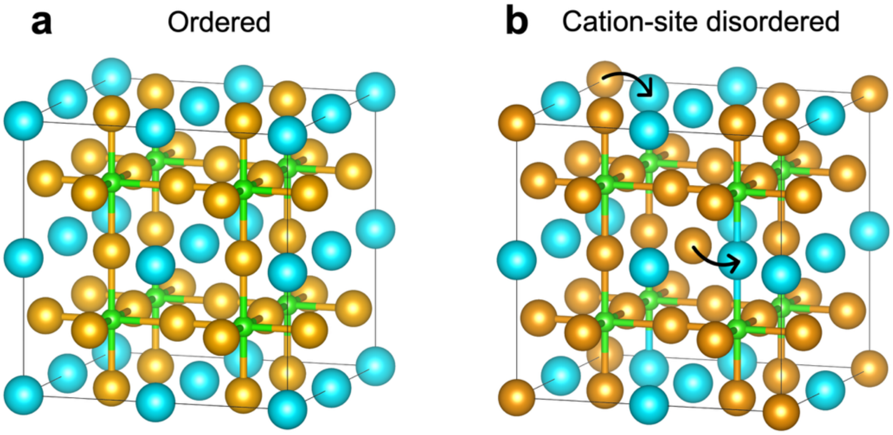


**Figure S14: Ordered and cation-site disordered 2x2x2 supercell of $Cu_3PdN$. a** Ordered anti-perovskite supercell. **b** Disordered supercell in which two Pd atoms are interchanged with adjacent Cu atoms, which amounts to 25% disorder.

**Beam Damage Study**

The beam damage study is carried out before starting the actual in situ measurements. Figure S14 shows the XANES spectra of the precursor solution measured at room temperature. When we expose the sample with x-ray at the same spot, we observe the sample is reduced after ~90 seconds. Thus, we measure 4 scans (20 sec/scan) at the same spot and after that we moved to the new spot on the sample. In this way, we avoid the beam damage, and all the in situ scans are carried at the same condition.

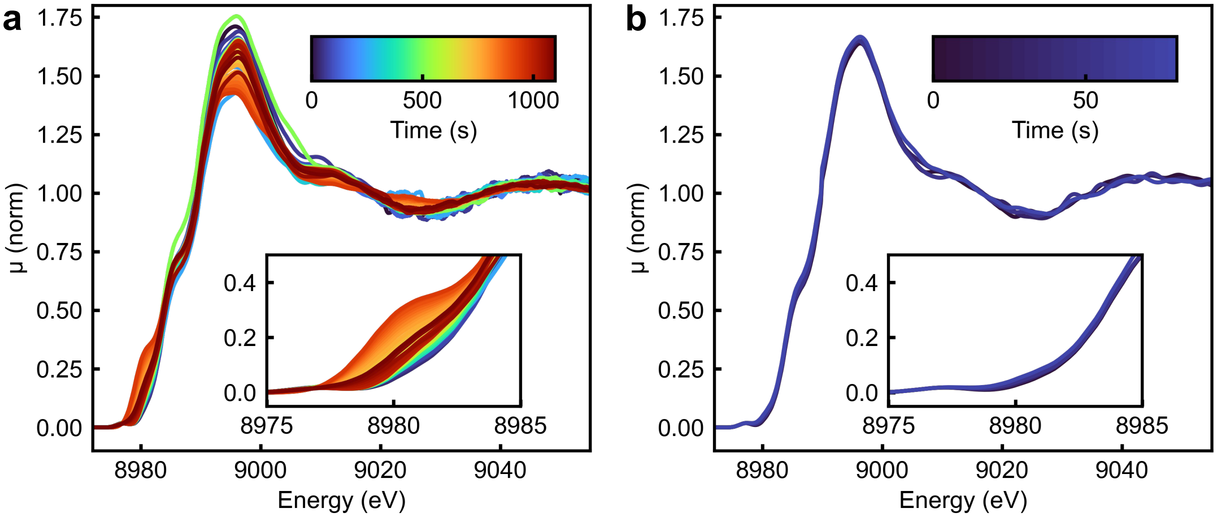


**Figure S15. Beam damage study on $Cu_3PdN$ precursor solution measured at room temperature (20 s/scan).** Fifty-five spectra measured at the same spot. **b** Four spectra measured at the same spot.

**Technical notes on PDF analysis of TS**

The in situ total scattering (TS) data for pair distribution function (PDF) analysis were acquired every 1 s using 2D detectors. The 2D scattering images were azimuthally integrated to 1D with PyFAI.[20] Consequently, the integrated scattering patterns were averaged to 60. The 1D scattering patterns are eventually processed to PDFs using PDFgetX3.[21] Table S6 gives the values used to process and Fourier transform the scattering patterns to the PDF.

**Table S6: Values used for processing the PDF.**

| | $q_{min}$ (Å$^{-1}$) | $q_{max}$ (Å$^{-1}$) | $q_{max,inst}$ (Å$^{-1}$) | $r_{poly}$ |
|---|---|---|---|---|
| **In situ PDF: Figure 3e, S15, S17** | 0.4 | 10.3 | 17.5 | 0.9 |
| **In situ PDF: Figure S18-20** | 0.7 | 16.3 | 18.3 | 0.9 |

The in situ PDF data shown in Figure 3e, S15, S17 focusses on the early stages of the reaction, which shows the NP nucleation and precursor conversion to the nitride, while the in situ PDF data shown in Figure S18-20 focusses on the growth of the NPs and the phase transition of $Cu_3PdN$ to $Cu_3Pd$. Due to the low scattering from the non-crystalline species present at the early stage of the reaction and a very high background contribution of the sample environment to the scattering signal, the PDF shown in Figure 3e, S15, S17 is obtained by using a low upper cut-off frequency $q_{max}$ which sacrifices $r$-resolution but reduces noise in the PDF data. The crystalline materials at the later stages of the reaction allow for a higher upper cut-off frequency $q_{max}$ without increasing the noise level in the PDF, as is used in Figure S18-20. Moreover, different background subtraction approaches have proven most reliable for the early-stage and late-stage PDF data sets, as explained below.

The background scattering images are acquired by measuring the same sample environment but without the addition of the precursor salts to the reaction solution at the same temperature ramping and processing the background scattering images as explained above. The background data was then subtracted from the data of the same temperature. Two different approaches are used to set the scale for background subtraction of the TS data. The data used in Figure 3e, S15, S17, is background subtracted by maximising the maximum intensity of the PDF between r = 1.9 and 4.0 Å. The data used for Figure S18-20 is background subtracted by fitting a Gaussian with a linear baseline to the glass peak at ~1.6 Å and reducing the amplitude using the following function:

$$y = a \cdot exp\left(\frac{-(x - x0)^2}{2 \cdot \left(\frac{FWHM}{2.35482}\right)^2}\right) + m \cdot x + y0 \quad (5)$$

*x0* is the position, *a* is the amplitude and *FWHM* is the full width at half maximum of the peak. *m* is the slope and *y0* is the intersection of the baseline with the y-axis. Table S7 shows the restraints used for the fitting.

**Table S7: Boundaries used for Gaussian fit as shown in Equation 5.**

| Parameter | Lower boundary | Upper boundary |
|---|---|---|
| **x0 (Å)** | 1.5 | 1.7 |
| **a (Å)** | 0.0 | ∞ |
| **FWHM (Å)** | 0.3 | 0.8 |
| **m ($Å^{-3}$)** | -1.0 | 1.0 |
| **y0 ($Å^{-2}$)** | - | - |

The in situ PDF data was refined using the diffpy-CMI library. The refinement was carried out sequentially, meaning the refined value of a parameter was used as the initial value for the refinement of the next time step. Table S8 shows the parameters used for the refinement, their restraints and the initial value for the refinement of the first time step. The refinement algorithm is shown in Table S9. N ADP is fixed to 0.35 $Å^{-2}$ throughout the refinement due to its strong correlation to the N occupancy. $q_{damp}$ and $q_{broad}$ are determined to 0.0281 and 0.0222 $Å^{-1}$, respectively, by refining the PDF of a $LaB_6$ standard filled in the heating cell.

**Table S8: Initial values and boundaries of PDF refinement parameter.**

| Parameter | Initial value | Lower boundary | Upper boundary |
|---|---|---|---|
| **Scale** | 0.3 | 0.0 | - |
| **Lattice parameter (Å)** | 3.84 | - | - |
| **Spherical domain size (Å)** | 30 | 15 | - |
| **N occupancy** | 1.0 | 0.0 | 1.0 |

| | | | |
|---|---|---|---|
| **$\partial_2$ ($Å^{-2}$)** | 3.5 | 1.5 | 10.0 |
| **N $U_{iso}$ ($Å^{-2}$)** | 0.35 | 0.35 | 0.35 |
| **Cu $U_{11}$ ($Å^{-2}$)** | 0.05 | 0.0001 | 0.5 |
| **Cu $U_{22}$ ($Å^{-2}$)** | 0.05 | 0.0001 | 0.5 |
| **Pd $U_{iso}$ ($Å^{-2}$)** | 0.05 | 0.0001 | 0.5 |

**Table S9: PDF refinement algorithm.**

| Refinement step | Included parameter for refinement |
|---|---|
| **1** | Scale |
| **2** | Scale, Particle size |
| **3** | Scale, Lattice parameter |
| **4** | Scale, N occupancy |
| **5** | Scale, Cu $U_{11}$, Cu $U_{22}$, Pd $U_{iso}$ |
| **6** | Scale, $\partial_2$ |
| **7** | Scale, Particle size, Lattice parameter, N occupancy, Cu $U_{11}$, Cu $U_{22}$, Pd $U_{iso}$, $\partial_2$ |

The code for the processing of the PDF data, including azimuthal integration of detector images, background subtraction approaches, averaging and Fourier transforming, as well as PDF refinement procedures, is available at:
https://gitlab.rrz.uni-hamburg.de/BAS0906/shm_2024_cu3pdn.

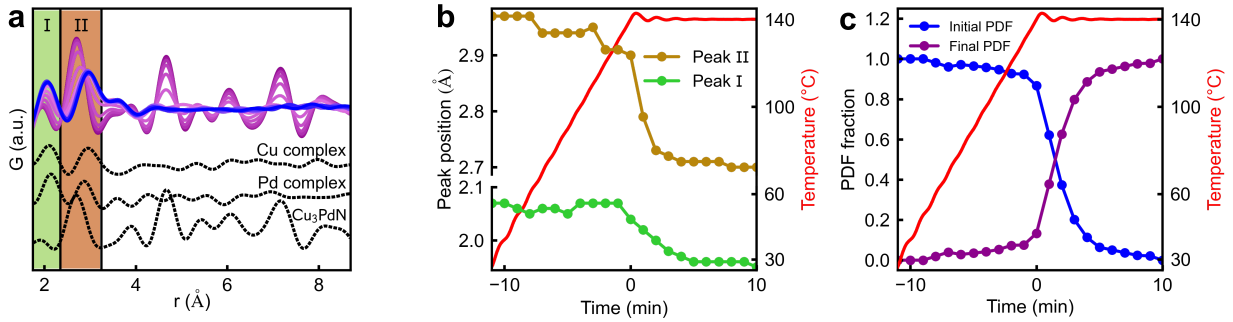


**Figure S16: In situ PDF analysis of the reaction to $Cu_3PdN$. a** In situ PDFs G(r). The in situ PDF data is compared to PDF simulations of the Cu and Pd starting complexes and $Cu_3PdN$ phase in dashed black traces. The first peak, peak I, highlighted in green, comprises the nearest neighbour interatomic distances Cu – N, Cu – O, Pd – N of the starting complexes as well as Cu – N of $Cu_3PdN$. The second peak, peak II, highlighted in orange, comprises the second neighbour interatomic distances Cu – Cu, Cu – C, and Pd – C of the starting complexes as well as Cu – Cu and Cu – Pd distances of $Cu_3PdN$ nanoparticles. Purely organic pair interatomic distances are not listed due to their weak scattering and weak contributions to the PDF signal. **b** Peak position of the first and second peak versus the reaction time. **c** PDF fraction of the linear combination of the initial PDF at t = -11 min and the last PDF at t = 10 min versus the reaction time.

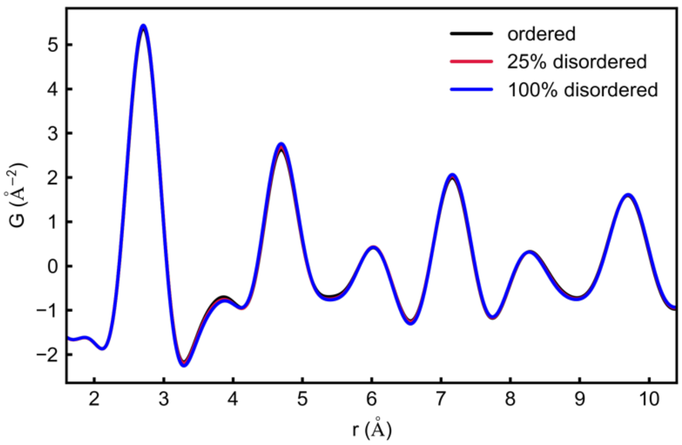


**Figure S17: PDF simulation comparison of ordered and disordered $Cu_3PdN$.** The ordered structure is the ideal anti-perovskite structure of $Cu_3PdN$ without cation-disorder. In the 25% disordered structure, 25 % of the Pd atoms interchange their position with 8 % of the Cu atoms to account for the stoichiometry of $Cu_3PdN$ (25 % / 3 ≈ 8 %). In the 100 % disordered structure, all Pd atoms interchange their position with 33 % of the Cu atoms. The parameters used in the PDF simulations are the same as the initial values for the PDF refinements, as listed in Table S8.

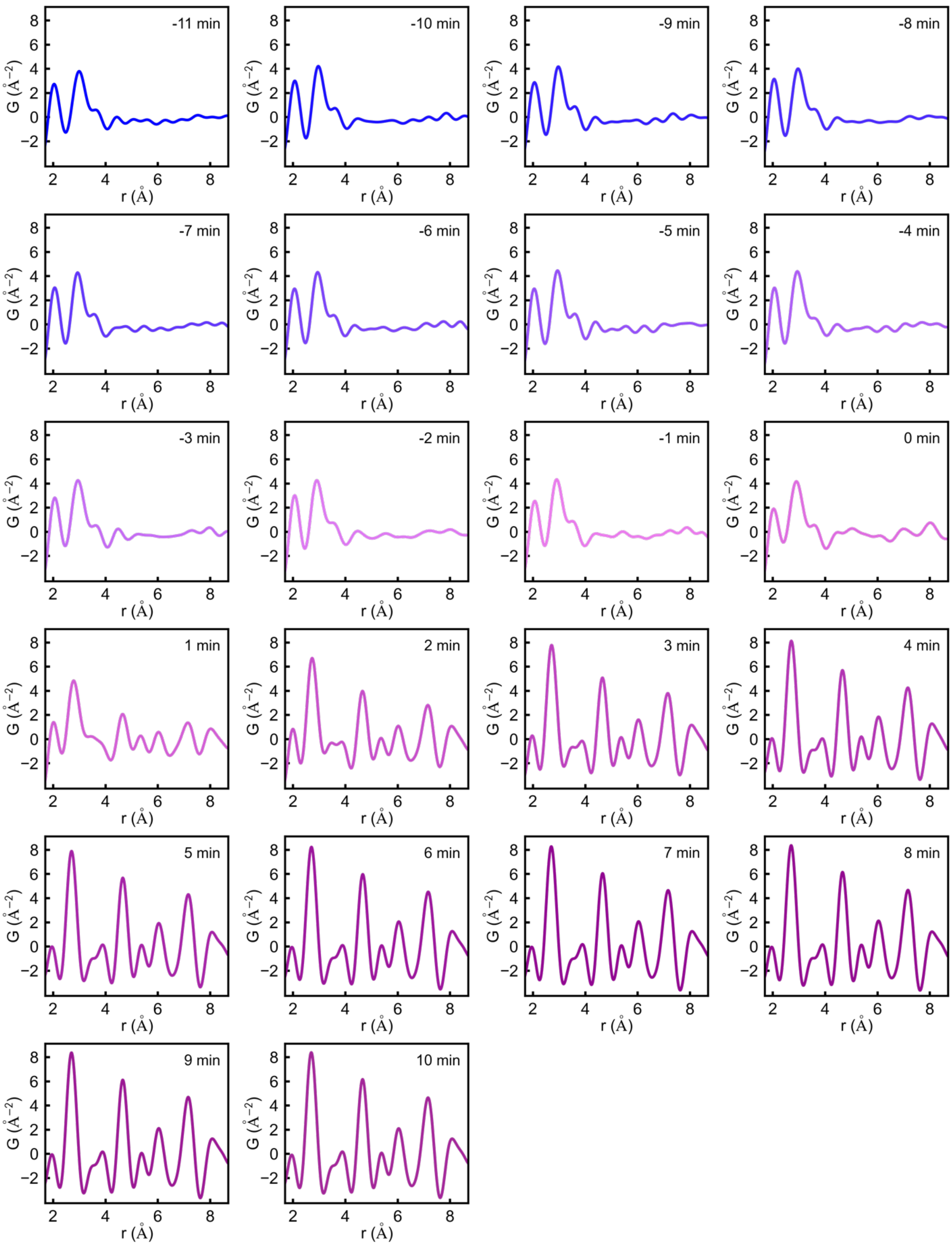


**Figure S18: Time series of PDFs, which are shown in Figure 3e and Figure S15.**

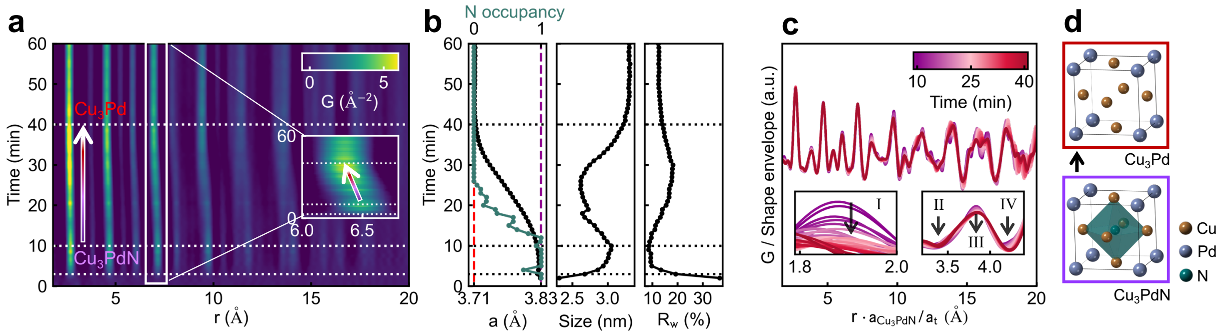


**Figure S19: In situ PDF of the reduction of $Cu_3PdN$ to bimetallic $Cu_3Pd$ at advanced reaction times. a** In situ time-resolved PDFs G(r). The different regions are highlighted by horizontal dashed lines. At ~3 min after reaching 140°C, $Cu_3PdN$ nanocrystals are emerging. The phase transformation takes place between ~10 and ~40 min. $Cu_3PdN$ and $Cu_3Pd$ have similar crystal structures and hence very similar PDFs. The phase transformation is mainly visible by a peak shift to lower r, which is due to the decrease of the lattice parameter when N leaves the crystal lattice. The inset shows a zoom of the peak at ~6.5 Å. **b** PDF refinement results. The phase transformation can be refined by using only the $Cu_3PdN$ phase and freeing the N occupancy. The first panel shows the decrease of the refined N occupancy and the lattice parameter, a. The second panel shows the refined spherical domain size. The third panel shows the goodness of the fit parameter $R_w$. **c** Overlay of normalized PDFs during the phase transformation. To account for the peak shifts during the phase transformation, each PDF x-axis is normalized by the respective refined lattice parameter $a_t$. To account for the change of intensity dampening due to the change of the domain size each PDF is normalized by the respective refined shape envelope. The insets zoom on selected N-Cu (I,IV), N-N (III), N-Pd (II) distances. The decrease of the intensities at the respective distances can be attributed to vanishing N. **d** Unit cells of $Cu_3PdN$ and $Cu_3Pd$.

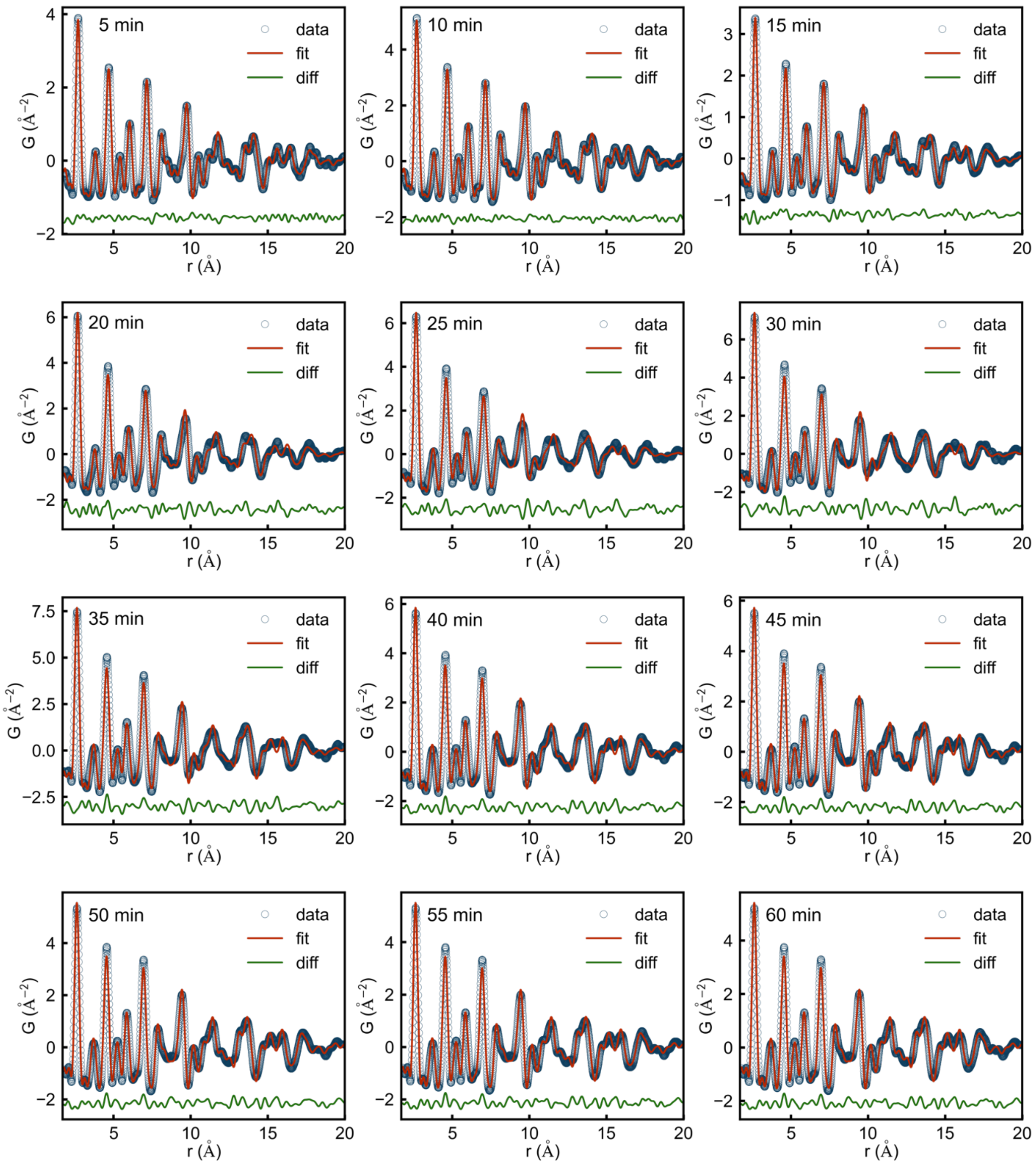


**Figure S20: Time series of PDF refinements 5 to 60 minutes reaction time at 140 °C.**

Refined parameters are shown in Figure S20.

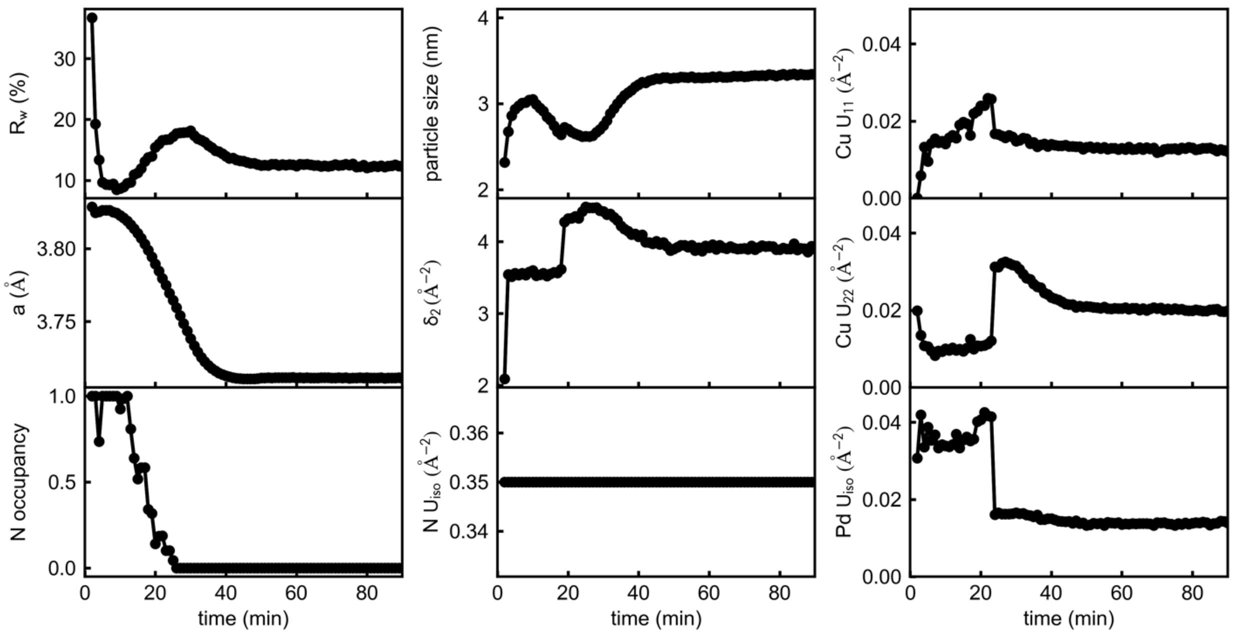


**Figure S21: Detailed representation of refined parameters of PDF refinements shown in Figure S19 and S20.**

## Electrocatalysis of HER

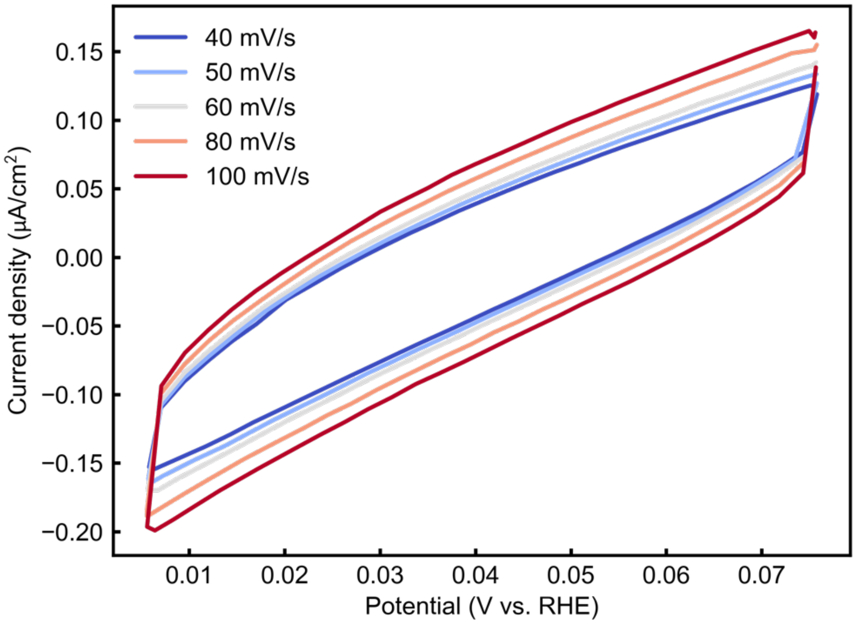


**Figure S22: Cyclic voltammetry measurement.**

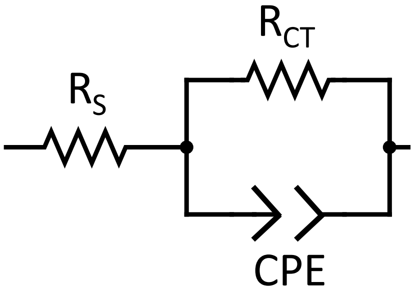


**Figure S23: Representation of the simple equivalent circle used to fit the EIS data.** $R_S$: solution resistance, $R_{CT}$: charge transfer resistance, CPE: constant phase element.

*Stability of $Cu_3PdN$ during EC*

First, we test the stability of $Cu_3PdN$ in the electrolyte. Therefore, 10 mg of $Cu_3PdN$ NPs are dispersed in 5 ml of 0.5M $H_2SO_4$ electrolyte for 20 h. Consequently, the $Cu_3PdN$ is washed and centrifuged 3 times with water, and 3 times with ethanol for 5 min at 10000 rpm. Finally, the product is dried under nitrogen flow and measured by PXRD. Figure S24 shows the PXRD pattern

that confirms the presence of anti-perovskite nitride phase without any additional bimetallic, metallic or oxide crystalline phases.

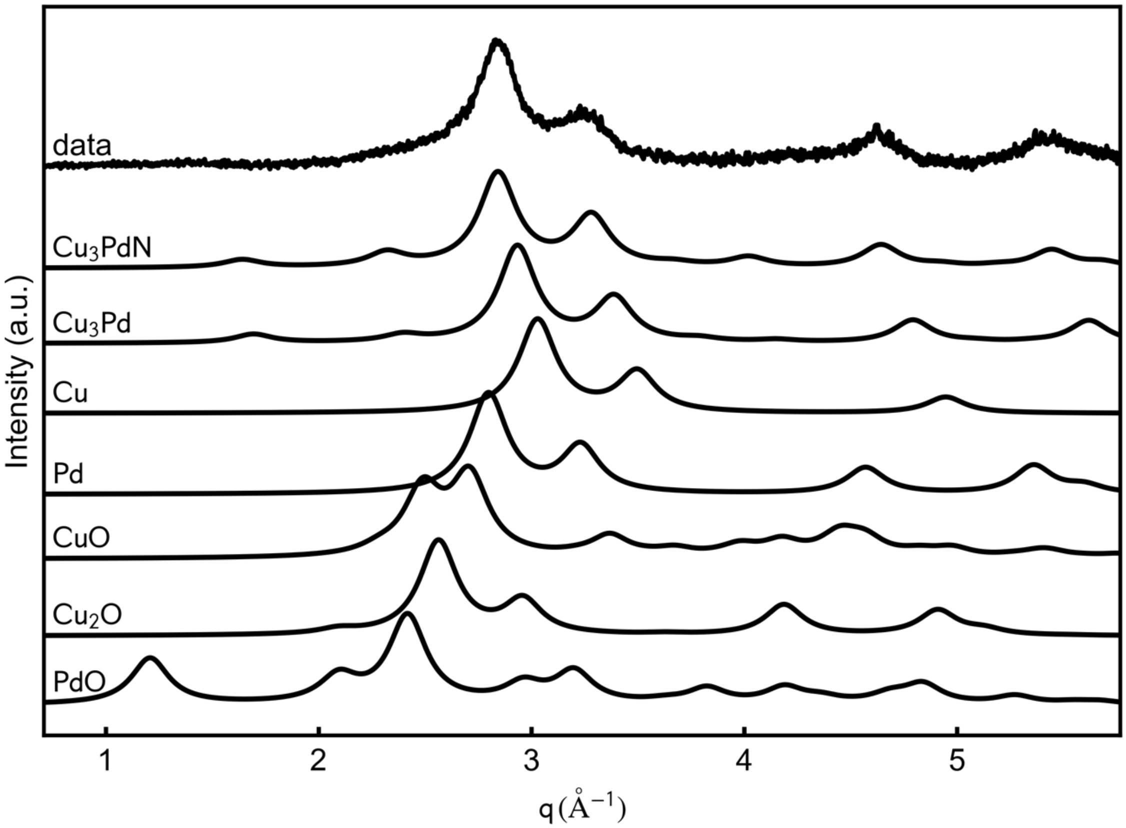


**Figure S24: PXRD of $Cu_3PdN$ after being dispersed in 0.5M $H_2SO_4$ electrolyte.** The experimental data is compared to PXRD simulations of $Cu_3PdN$, $Cu_3Pd$, Cu, Pd, CuO, $Cu_2O$ and PdO. All patterns are simulated using GSASII[8] and assuming a crystallite size of 3 nm.

Further, we test the stability of $Cu_3PdN$ by comparing XPS before and after EC. Figure S25 shows Cu 2p, Pd 3d, N 1s and O 1s XP spectra before and after EC. The pristine sample is measured on a carbon tape. XPS after EC is directly measured on the GC working electrode. Despite the low intensity compared to the pristine sample due to the very low concentration of the sample deposited (300 - 400 μg) on the GC electrode, all the features are the same as the pristine sample, confirming the stability of the $Cu_3PdN$ during the electrochemical measurement. However, the Cu 2p spectral feature is negatively shifted by about 0.7 eV, and the corresponding N 1s is positively shifted by 1.2 eV, which could be due to the low charge density of Cu compared to the pristine sample. Furthermore, the O 1s core-level spectrum is deconvoluted into 3 obvious peaks related to metal-

oxygen, OH, and COOH contributions as in the pristine sample. Together with the presence of Cu(II) and Pd(II), it is clear that a thin layer of amorphous metal oxide and hydroxide is formed on the surface of nitride NPs.[1] Despite this, it further proves that the $Cu_3PdN$ NPs are highly stable during the electrochemical measurements.

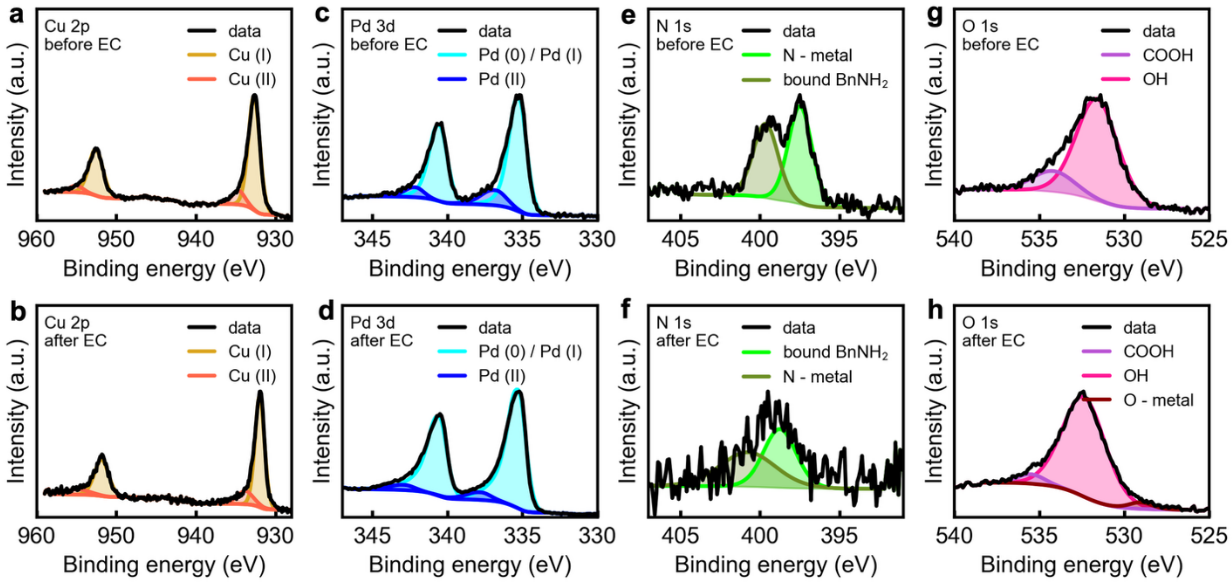


**Figure S25: XPS core-level spectra of $Cu_3PdN$ before and after EC.** Cu 2p **a** before and **b** after EC, Pd 3d **c** before and **d** after EC, N 1s **e** before and **f** after EC, O 1s **g** before and **h** after EC.

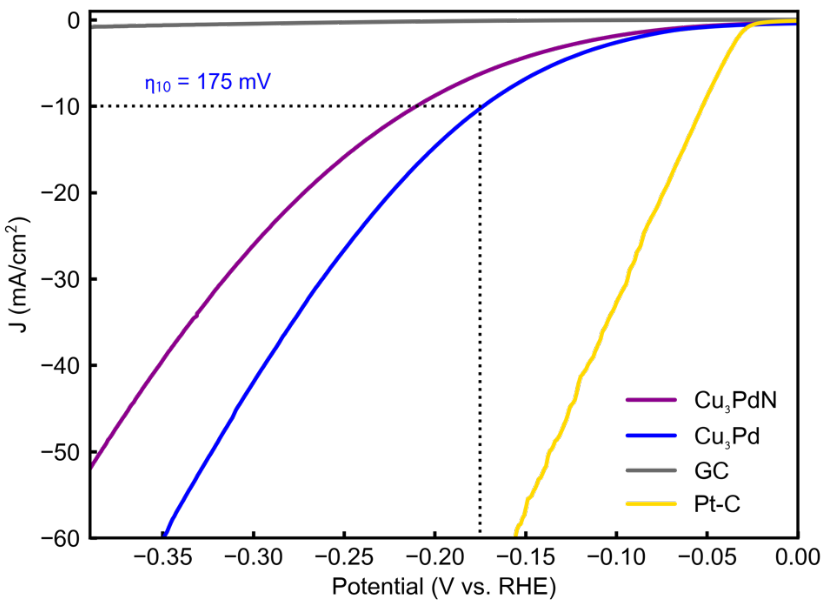


**Figure S26: LSV of $Cu_3Pd$ compared to $Cu_3PdN$, Pt-C and GC, showing an overpotential of 175 mV.**

**Table S10: Overview of TMN-based electrocatalysts for HER.** GC: glassy carbon, CC: carbon cloth, rGO: reduced graphene oxide, NC: N-doped carbon, NG: N-doped graphene, n.a.: not available.

| Material | Substrate | Medium | $\eta_{10}$ (mV) | Tafel slope (mV/dec) | Reference |
|---|---|---|---|---|---|
| **$Co_{0.6}Mo_{1.4}N_2$** | GC | 0.1 M HClO4 | 190 | n.a. | [22] |
| **$CoFeN_x$** | Ni-foam | 1 M KOH | 23 | 94 | [23] |
| **CoN-Co** | Co-foam | 1 M KOH | 12 | 41.6 | [24] |
| **$Cu_3PdN$** | GC | 0.5 M $H_2SO_4$ | 212 | 122 | This work |
| **GaN** | sapphire | 0.5 M $H_2SO_4$ | 168 | 35 | [25] |
| **$Mo_2N$** | GC | 1 M KOH | 353 | 108 | [26] |
| **$Mo_2N$- $Mo_2C$** | GC | 0.5 M $H_2SO_4$ | 205 | 72 | [27] |
| **$Mo_2N$- $Mo_2C$-NC** | GC | 0.5 M $H_2SO_4$ | 185 | 56 | [28] |
| **$Mo_2N$- $Mo_2C$-rGO** | GC | 0.5 M $H_2SO_4$ | 157 | 55 | [29] |
| **$Mo_2N$-NC** | GC | 0.5 M $H_2SO_4$ | 217 | 116 | [30] |
| **MoN** | GC | 0.1 M $HClO_4$ | 420 | n.a. | [22] |
| **$Ni_2N$** | Ni-foam | 1 M KOH | 55 | 54 | [31] |
| **$Ni_3FeN$** | CC | 1 M KOH | 238 | 46 | [32] |
| **$Ni_3FeN$** | GC | 1 M KOH | 158 | 46 | [33] |
| **$Ni_3N$** | Ti mesh | 0.5M K-Bi | 297 | 165 | [34] |
| **$Ni_3N$-Ni-Pt-C** | GC | 0.5 M $H_2SO_4$ | 117 | 47 | [35] |
| **NiCoN** | CC | 1 M NaOH | 145 | 105 | [36] |

| | | | | | |
|---|---|---|---|---|---|
| **NiCoN-C** | GC | 1 M KOH | 103 | n.a. | [37] |
| **Pt-C** | GC | 0.5 M $H_2SO_4$ | 53 | 17 | This work |
| **TiN-NG** | GC | 0.5 M $H_2SO_4$ | 161 | 66 | [38] |
| **TiN-Pt** | CC | 0.5M $H_2SO_4$ | 40 | 39 | [39] |
| **VCoN** | GC | 1 M KOH | 179 | 123 | [40] |
| **VMoN** | CC | 0.5 M $H_2SO_4$ | 108 | 60 | [41] |
| **VN** | GC | 1 M KOH | 476 | 165 | [40] |
| **VN-Co-P** | GC | 1 M KOH | 137 | 81 | [42] |
| **VN-Co-P** | GC | 1 M KOH | 137 | 81 | [42] |
| **WCN-Fe** | GC | 0.5M $H_2SO_4$ | 220 | 47 | [43] |
| **WN** | CC | 0.5M $H_2SO_4$ | 198 | 92 | [44] |
| **-** | GC | 0.5M $H_2SO_4$ | 768 | 366 | This work |

**References**


[1] M. Parvizian, A. Duràn Balsa, R. Pokratath, C. Kalha, S. Lee, D. Van den Eynden, M. Ibáñez, A. Regoutz, J. De Roo, The Chemistry of Cu3N and Cu3PdN Nanocrystals**, *Angewandte Chemie International Edition*, 2022, 61, e202207013, https://doi.org/10.1002/anie.202207013.

[2] K. Yao, Z. Fang, J. Wang, W. Wang, M. Wang, W. Yan, M. Ye, B. Jiang, K. Wu, X. Wei, Regulating charge distribution of Cu $_3$ PdN nanocrystals for nitrate electroreduction to ammonia, *Chemical Communications*, 2023, 59, 12176, https://doi.org/10.1039/D3CC02791K.

[3] J. Jia, X. Hao, Y. Chang, M. Jia, Z. Wen, Rational design of Cu3PdN nanocrystals for selective electroreduction of carbon dioxide to formic acid, *Journal of Colloid and Interface Science*, 2021, 586, 491, https://doi.org/10.1016/j.jcis.2020.10.112.

[4] R. W. Lord, C. F. Holder, J. L. Fenton, R. E. Schaak, Seeded Growth of Metal Nitrides on Noble-Metal Nanoparticles To Form Complex Nanoscale Heterostructures, *Chemistry of Materials*, 2019, 31, 4605, https://doi.org/10.1021/acs.chemmater.9b01638.

[5] J. Jia, M. Shao, G. Wang, W. Deng, Z. Wen, Cu 3 PdN nanocrystals electrocatalyst for formic acid oxidation, *Electrochemistry Communications*, 2016, 71, 61, https://doi.org/10.1016/j.elecom.2016.08.009.

[6] D. D. Vaughn II, J. Araujo, P. Meduri, J. F. Callejas, M. A. Hickner, R. E. Schaak, Solution Synthesis of Cu $_3$ PdN Nanocrystals as Ternary Metal Nitride Electrocatalysts for the Oxygen Reduction Reaction, *Chemistry of Materials*, 2014, 26, 6226, https://doi.org/10.1021/cm5029723.

[7] T. Li, J. Wang, S. Zhu, E. P. Delmo, F. Sun, L. Zhang, M. Gu, M. Shao, Cu3PdxN nanocrystals for efficient CO2 electrochemical reduction to methane, *Electrochimica Acta*, 2021, 371, 137793, https://doi.org/10.1016/j.electacta.2021.137793.

[8] B. H. Toby, R. B. Von Dreele, GSAS-II: the genesis of a modern open-source all purpose crystallography software package, *Journal of Applied Crystallography*, 2013, 46, 544, https://doi.org/10.1107/S0021889813003531.

[9] T. Egami, S. J. L. Billinge, Underneath the Bragg Peaks: Structural Analysis of Complex Materials, 2012, 16, https://books.google.de/books?id=kzeSEdYS54wC.

[10] V. A. Solé, E. Papillon, M. Cotte, Ph. Walter, J. Susini, A multiplatform code for the analysis of energy-dispersive X-ray fluorescence spectra, *Spectrochimica Acta Part B: Atomic Spectroscopy*, 2007, 62, 63, https://doi.org/10.1016/j.sab.2006.12.002.

[11] J. A. Reuter, M. Menzel, SLIX: A Python package for fully automated evaluation of Scattered Light Imaging measurements on brain tissue, *Journal of Open Source Software*, 2020, 5, 2675, https://doi.org/10.21105/joss.02675.

[12] P. H. C. Eilers, A Perfect Smoother, *Analytical Chemistry*, 2003, 75, 3631, https://doi.org/10.1021/ac034173t.

[13] C. R. Harris, K. J. Millman, S. J. van der Walt, R. Gommers, P. Virtanen, D. Cournapeau, E. Wieser, J. Taylor, S. Berg, N. J. Smith, R. Kern, M. Picus, S. Hoyer, M. H. van Kerkwijk, M. Brett, A. Haldane, J. F. Del Río, M. Wiebe, P. Peterson, P. Gérard-Marchant, K. Sheppard, T. Reddy, W. Weckesser, H. Abbasi, C. Gohlke, T. E. Oliphant, Array programming with NumPy, *Nature*, 2020, 585, 357, https://doi.org/10.1038/s41586-020-2649-2.

[14] P. Virtanen, R. Gommers, T. E. Oliphant, M. Haberland, T. Reddy, D. Cournapeau, E. Burovski, P. Peterson, W. Weckesser, J. Bright, S. J. van der Walt, M. Brett, J. Wilson, K. J. Millman, N. Mayorov, A. R. J. Nelson, E. Jones, R. Kern, E. Larson, C. J. Carey, İ. Polat, Y. Feng, E. W. Moore, J. VanderPlas, D. Laxalde, J. Perktold, R. Cimrman, I. Henriksen, E. A. Quintero, C. R. Harris, A. M. Archibald, A. H. Ribeiro, F. Pedregosa, P. van Mulbregt, SciPy 1.0: fundamental algorithms for scientific computing in Python, *Nature Methods*, 2020, 17, 261, https://doi.org/10.1038/s41592-019-0686-2.

[15] B. Ravel, M. Newville, ATHENA, ARTEMIS, HEPHAESTUS: data analysis for X-ray absorption spectroscopy using IFEFFIT, *Journal of Synchrotron Radiation*, 2005, 12, 537, https://doi.org/10.1107/S0909049505012719.

[16] A. de Juan, J. Jaumot, R. Tauler, Multivariate Curve Resolution (MCR). Solving the mixture analysis problem, *Analytical Methods*, 2014, 6, 4964, https://doi.org/10.1039/C4AY00571F.

[17] J. Jaumot, R. Gargallo, A. de Juan, R. Tauler, A graphical user-friendly interface for MCR-ALS: a new tool for multivariate curve resolution in MATLAB, *Chemometrics and Intelligent Laboratory Systems*, 2005, 76, 101, https://doi.org/10.1016/j.chemolab.2004.12.007.

[18] J. Jaumot, A. de Juan, R. Tauler, MCR-ALS GUI 2.0: New features and applications, *Chemometrics and Intelligent Laboratory Systems*, 2015, 140, 1, https://doi.org/10.1016/j.chemolab.2014.10.003.

[19] W. Windig, D. A. Stephenson, Self-modeling mixture analysis of second-derivative near-infrared spectral data using the SIMPLISMA approach, *Analytical Chemistry*, 1992, 64, 2735, https://doi.org/10.1021/ac00046a015.

[20] G. Ashiotis, A. Deschildre, Z. Nawaz, J. P. Wright, D. Karkoulis, F. E. Picca, J. Kieffer, The fast azimuthal integration Python library: pyFAI, *Journal of Applied Crystallography*, 2015, 48, 510, https://doi.org/10.1107/S1600576715004306.

[21] P. Juhás, T. Davis, C. L. Farrow, S. J. L. Billinge, PDFgetX3: A rapid and highly automatable program for processing powder diffraction data into total scattering pair distribution functions, *J. Appl. Crystallogr.*, 2013, 46, 560, https://doi.org/10.1107/S0021889813005190.

[22] B. Cao, G. M. Veith, J. C. Neuefeind, R. R. Adzic, P. G. Khalifah, Mixed Close-Packed Cobalt Molybdenum Nitrides as Non-noble Metal Electrocatalysts for the Hydrogen Evolution Reaction, *Journal of the American Chemical Society*, 2013, 135, 19186, https://doi.org/10.1021/ja4081056.

[23] Y. Wang, D. Liu, Z. Liu, C. Xie, J. Huo, S. Wang, Porous cobalt-iron nitride nanowires as excellent bifunctional electrocatalysts for overall water splitting, *Chemical Communications*, 2016, 52, 12614, https://doi.org/10.1039/c6cc06608a.

[24] F. Song, W. Li, J. Yang, G. Han, T. Yan, X. Liu, Y. Rao, P. Liao, Z. Cao, Y. Sun, Interfacial Sites between Cobalt Nitride and Cobalt Act as Bifunctional Catalysts for Hydrogen Electrochemistry, *ACS Energy Letters*, 2019, 4, 1594, https://doi.org/10.1021/acsenergylett.9b00738.

[25] H. Hu, B. Chang, X. Sun, Q. Huo, B. Zhang, Y. Li, Y. Shao, L. Zhang, Y. Wu, X. Hao, Intrinsic Properties of Macroscopically Tuned Gallium Nitride Single-Crystalline Facets for Electrocatalytic Hydrogen Evolution, *Chemistry – A European Journal*, 2019, 25, 10420, https://doi.org/10.1002/chem.201901395.

[26] L. Ma, L. R. L. Ting, V. Molinari, C. Giordano, B. S. Yeo, Efficient hydrogen evolution reaction catalyzed by molybdenum carbide and molybdenum nitride nanocatalysts synthesized via the urea glass route, *Journal of Materials Chemistry A*, 2015, 3, 8361, https://doi.org/10.1039/C5TA00139K.
[27] W. Wang, C. Liu, D. Zhou, L. Yang, J. Zhou, D. Yang, In-situ synthesis of coupled molybdenum carbide and molybdenum nitride as electrocatalyst for hydrogen evolution reaction, *Journal of Alloys and Compounds*, 2019, 792, 230, https://doi.org/10.1016/j.jallcom.2019.03.397.
[28] R. Cheng, H. He, Z. Pu, I. S. Amiinu, L. Chen, Z. Wang, G. Li, S. Mu, Shrunken hollow Mo-N/Mo-C nanosphere structure for efficient hydrogen evolution in a broad pH range, *Electrochimica Acta*, 2019, 298, 799, https://doi.org/10.1016/j.electacta.2018.12.128.
[29] H. Yan, Y. Xie, Y. Jiao, A. Wu, C. Tian, X. Zhang, L. Wang, H. Fu, Holey Reduced Graphene Oxide Coupled with an Mo2N–Mo2C Heterojunction for Efficient Hydrogen Evolution, *Advanced Materials*, 2018, 30, 1704156, https://doi.org/10.1002/adma.201704156.
[30] Y.-J. Song, Z.-Y. Yuan, One-pot Synthesis of Mo2N/NC Catalysts with Enhanced Electrocatalytic Activity for Hydrogen Evolution Reaction, *Electrochimica Acta*, 2017, 246, 536, https://doi.org/10.1016/j.electacta.2017.06.086.
[31] Z. Ma, Z. Li, S. Li, P. Li, H. Zhang, Nanostructured Ni2N thin films magnetron-sputtered on nickel foam as efficient electrocatalyst for hydrogen evolution reaction, *Materials Letters*, 2018, 229, 148, https://doi.org/10.1016/j.matlet.2018.07.016.
[32] Q. Chen, R. Wang, M. Yu, Y. Zeng, F. Lu, X. Kuang, X. Lu, Bifunctional Iron–Nickel Nitride Nanoparticles as Flexible and Robust Electrode for Overall Water Splitting, *Electrochimica Acta*, 2017, 247, 666, https://doi.org/10.1016/j.electacta.2017.07.025.
[33] X. Jia, Y. Zhao, G. Chen, L. Shang, R. Shi, X. Kang, G. I. N. Waterhouse, L.-Z. Wu, C.-H. Tung, T. Zhang, Ni3FeN Nanoparticles Derived from Ultrathin NiFe-Layered Double Hydroxide Nanosheets: An Efficient Overall Water Splitting Electrocatalyst, *Advanced Energy Materials*, 2016, 6, 1502585, https://doi.org/10.1002/aenm.201502585.
[34] L. Xie, F. Qu, Z. Liu, X. Ren, S. Hao, R. Ge, G. Du, A. M. Asiri, X. Sun, L. Chen, In situ formation of a 3D core/shell structured $Ni_3$ N@Ni–Bi nanosheet array: an efficient non-noble-metal bifunctional electrocatalyst toward full water splitting under near-neutral conditions, *Journal of Materials Chemistry A*, 2017, 5, 7806, https://doi.org/10.1039/C7TA02333B.
[35] C. Wang, Y. Sun, E. Tian, D. Fu, M. Zhang, X. Zhao, W. Ye, Easy access to trace-loading of Pt on inert Ni3N nanoparticles with significantly improved hydrogen evolution activity at entire pH values, *Electrochimica Acta*, 2019, 320, 134597, https://doi.org/10.1016/j.electacta.2019.134597.
[36] L. Han, K. Feng, Z. Chen, Self-Supported Cobalt Nickel Nitride Nanowires Electrode for Overall Electrochemical Water Splitting, *Energy Technology*, 2017, 5, 1908, https://doi.org/10.1002/ente.201700108.
[37] J. Lai, B. Huang, Y. Chao, X. Chen, S. Guo, Strongly Coupled Nickel–Cobalt Nitrides/Carbon Hybrid Nanocages with Pt-Like Activity for Hydrogen Evolution Catalysis, *Advanced Materials*, 2019, 31, 1805541, https://doi.org/10.1002/adma.201805541.
[38] G. S. Shanker, G. B. Markad, M. Jagadeeswararao, U. Bansode, A. Nag, Colloidal Nanocomposite of TiN and N-Doped Few-Layer Graphene for Plasmonics and

Electrocatalysis, *ACS Energy Letters*, 2017, 2, 2251, https://doi.org/10.1021/acsenergylett.7b00741.
[39] C. Wang, H. Shi, H. Liu, J. Fu, D. Wei, W. Zeng, Q. Wan, G. Zhang, H. Duan, Quasi-atomic-scale platinum anchored on porous titanium nitride nanorod arrays for highly efficient hydrogen evolution, *Electrochimica Acta*, 2018, 292, 727, https://doi.org/10.1016/j.electacta.2018.10.011.
[40] N. Zhang, L. Cao, L. Feng, J. Huang, K. Kajiyoshi, C. Li, Q. Liu, D. Yang, J. He, Co,N-Codoped porous vanadium nitride nanoplates as superior bifunctional electrocatalysts for hydrogen evolution and oxygen reduction reactions, *Nanoscale*, 2019, 11, 11542.
[41] B. Wei, G. Tang, H. Liang, Z. Qi, D. Zhang, W. Hu, H. Shen, Z. Wang, Bimetallic vanadium-molybdenum nitrides using magnetron co-sputtering as alkaline hydrogen evolution catalyst, *Electrochemistry Communications*, 2018, 93, 166, https://doi.org/10.1016/j.elecom.2018.07.012.
[42] H. Yang, Y. Hu, D. Huang, T. Xiong, M. Li, M.-S. Balogun, Y. Tong, Efficient hydrogen and oxygen evolution electrocatalysis by cobalt and phosphorus dual-doped vanadium nitride nanowires, *Materials Today Chemistry*, 2019, 11, 1, https://doi.org/10.1016/j.mtchem.2018.10.004.
[43] Y. Zhao, K. Kamiya, K. Hashimoto, S. Nakanishi, Hydrogen Evolution by Tungsten Carbonitride Nanoelectrocatalysts Synthesized by the Formation of a Tungsten Acid/Polymer Hybrid In Situ, *Angewandte Chemie International Edition*, 2013, 52, 13638, https://doi.org/10.1002/anie.201307527.
[44] J. Shi, Z. Pu, Q. Liu, A. M. Asiri, J. Hu, X. Sun, Tungsten nitride nanorods array grown on carbon cloth as an efficient hydrogen evolution cathode at all pH values, *Electrochimica Acta*, 2015, 154, 345, https://doi.org/10.1016/j.electacta.2014.12.096.
